# Modeling Effects of Spatial Heterogeneities and Layered Exposure Interventions on the Spread of COVID-19 across New Jersey

**DOI:** 10.3390/ijerph182211950

**Published:** 2021-11-14

**Authors:** Xiang Ren, Clifford P. Weisel, Panos G. Georgopoulos

**Affiliations:** 1 Environmental and Occupational Health Sciences Institute (EOHSI), Rutgers University, Piscataway, NJ 08854, USA; xr32@scarletmail.rutgers.edu (X.R.); weisel@eohsi.rutgers.edu (C.P.W.); 2 Department of Chemical and Biochemical Engineering, Rutgers University, Piscataway, NJ 08854, USA; 3 Department of Environmental and Occupational Health and Justice, Rutgers School of Public Health, Piscataway, NJ 08854, USA; 4 Department of Environmental Sciences, Rutgers University, New Brunswick, NJ 08901, USA

**Keywords:** COVID-19, SARS-CoV-2, stochastic SEIR (Susceptible, Exposed, Infected, Recovered) model, SQMC (Sequential Quasi-Monte Carlo) parameters optimization, exposure factors, face-mask wearing, heterogeneities, first wave, New Jersey

## Abstract

COVID-19 created an unprecedented global public health crisis during 2020–2021. The severity of the fast-spreading infection, combined with uncertainties regarding the physical and biological processes affecting transmission of SARS-CoV-2, posed enormous challenges to healthcare systems. Pandemic dynamics exhibited complex spatial heterogeneities across multiple scales, as local demographic, socioeconomic, behavioral and environmental factors were modulating population exposures and susceptibilities. Before effective pharmacological interventions became available, controlling exposures to SARS-CoV-2 was the only public health option for mitigating the disease; therefore, models quantifying the impacts of heterogeneities and alternative exposure interventions on COVID-19 outcomes became essential tools informing policy development. This study used a stochastic SEIR framework, modeling each of the 21 New Jersey counties, to capture important heterogeneities of COVID-19 outcomes across the State. The models were calibrated using confirmed daily deaths and SQMC optimization and subsequently applied in predictive and exploratory modes. The predictions achieved good agreement between modeled and reported death data; counterfactual analysis was performed to assess the effectiveness of layered interventions on reducing exposures to SARS-CoV-2 and thereby fatality of COVID-19. The modeling analysis of the reduction in exposures to SARS-CoV-2 achieved through concurrent social distancing and face-mask wearing estimated that 357 [IQR (290, 429)] deaths per 100,000 people were averted.

## 1. Introduction

### 1.1. Background: Characterizing Pathways of COVID-19 Transmission

COVID-19 presents the greatest public health challenge humanity has faced in over a century. In 2020 it became the third leading cause of deaths in the US behind heart disease and cancer [1,2] and in the Fall of 2021 still remains a major threat to the nation’s and the world’s health and well-being. In the initial phases of the pandemic, before effective preventive and therapeutic pharmacological interventions became available, controlling population exposures to the novel coronavirus SARS-CoV-2 was the only public health option available for mitigating the disease. However, developing rational and effective exposure intervention strategies, especially during the first half of 2020, was substantially hindered by complexities and uncertainties associated with the physical and biological processes affecting the spread of the disease. On one hand, it was recognized quite early in the pandemic that SARS-CoV-2 is transmitted not only by symptomatic but also by presymptomatic and asymptomatic individuals [3,4]. On the other hand, in the initial phase of the pandemic there were significant uncertainties and disagreements regarding the relative importance of the various routes and pathways involved in viral transmission [5]. Droplet spray was initially thought to be the primary means of SARS-CoV-2 transmission [6]. Viral respiratory droplets are produced when an infected person sneezes, coughs, or talks; these droplets can be directly deposited on the noses, mouths, and eyes of people nearby (usually at distances < 2 m) and can cause an infection: this is typically referred to as “direct contact of transmission”. Direct/indirect contact provides another potential pathway, where people are exposed by touching surfaces carrying the virus, such as an infected person’s hand and other fomites, and then touching their own eyes, noses, and mouths.

The importance of aerosol inhalation in COVID-19 transmission was not fully recognized initially. In April 2020, Morawska and Cao [7] pointed out the following: “Hand washing and maintaining social distance are the main measures recommended by the World Health Organization (WHO) to avoid contracting COVID-19. Unfortunately, these measures do not prevent infection by inhalation of small droplets exhaled by an infected person that can travel distances of meters or tens of meters in the air and carry their viral content.” Almost three months later, in July 2020, a follow-up article by Morawska and Milton [8], was co-signed by 239 scientists and started with the statement: “We appeal to the medical community and to the relevant national and international bodies to recognize the potential for airborne spread of coronavirus disease 2019 (COVID-19). There is significant potential for inhalation exposure to viruses in microscopic respiratory droplets (microdroplets) at short to medium distances (up to several meters, or room scale), and we are advocating for the use of preventive measures to mitigate this route of airborne transmission.” This commentary was widely publicized and eventually agencies, such as the World Health Organization, adopted exposure mitigation measures that incorporated face-mask wearing. Aerosols (e.g., droplets or particles with effective diameters less than 5–10 µm) can contain infectious virions and remain suspended in air for hours [9,10,11], resulting in them being transported and inhaled by individuals at distances substantially beyond 1–2 m from the infected persons [12]. Aerosol-based transmission of SARS-CoV-2 has been explored in several laboratory studies [13,14,15], while extensive work is ongoing to further elucidate the role of this transmission mode on the spread of the COVID-19 pandemic [16,17,18]. A wide spectrum of studies reveal multiple physical and biological process complexities associated with the aerosol transmission pathway, ranging from the role of indoor ventilation patterns [19] to different viral loads in aerosols for different variants of SARS-CoV-2 [20,21].

It is now widely accepted [5,22,23] that all of the above pathways of transmission must be controlled in order to reduce exposures to SARS-CoV-2 and minimize the spread of COVID-19. To achieve this, a hierarchy of “layered controls” aiming to contain the emission of virus-containing droplets and aerosols, curtail inter-individual contact, and frequent cleaning/disinfection were recommended to the public as exposure-reducing interventions [5]. Wearing a face mask provides “source control” that reduces both emissions and intake of virus-laden droplets and aerosols; for example, N95 masks are personal protective equipment (PPE) for both the wearer and nearby individuals, while both disposable surgical masks and reusable cloth masks have been widely used around the world as “community protective equipment” (CPE). Physical distancing and spacing mitigate the pathways of direct contact, prevent large expired droplets from the infected individuals reaching other persons, and also reduce the risk of exposures to viral aerosols. It should be noted that people in proximity to the infected individuals are expected to be exposed to higher concentrations of virus-laden aerosols than those that are more distant. Ventilation and filtration are indoor environmental control strategies that can reduce the risk of aerosol inhalation by diluting/removing the virus-containing droplets and aerosols in the air. Cleaning and disinfection practices eliminate or reduce the viral load on fomites, mitigating direct/indirect contact, and also reduce the risk of exposure to infectious aerosols that can potentially be resuspended from fomites into the air.

Governing authorities of countries, regional entities, and cities instituted combinations of public health interventions to reduce general population exposures to SARS-CoV-2, including closures of schools and non-essential businesses, social distancing, wearing face masks, travel restrictions, testing, tracing and isolation of exposed individuals, lockdown of specific areas, etc. [24,25,26]. The effectiveness of these policies has varied substantially across different regions and has been closely related to how communities have complied with them. In reality, both person-to-person infectious disease transmission and the resulting severity of viral infection are complex processes that are driven by multiple biological, demographic/behavioral, and environmental factors. Horton [27] correctly stated that COVID-19 is not a pandemic but a “syndemic” in order to emphasize the importance of numerous biological, social and environmental determinants of health (e.g., sex, age, obesity, medical histories, occupation, prior exposures to chemical, physical, biological and psychosocial stressors, etc.). For instance, older individuals [28,29,30], particularly those residing in nursing homes [31], and males with preexisting conditions (e.g., metabolic disorders, such as diabetes; chronic lung conditions, such as moderate to severe asthma; cardiac diseases with complications, etc.) [32] have been far more vulnerable to COVID-19 than healthy younger individuals. Workers employed in essential services (e.g., medical facilities, food and medical supply chains, transportation infrastructure, government operations, security, etc.) face significantly higher risks of exposure to the virus than those who can work from home [33]. This has resulted to a significantly disproportionate impact of the disease on communities of color [34,35].

### 1.2. Modeling COVID-19: Computational Approaches

Computational models provide essential tools for framing our understanding of the complexity of disease dynamics, including underlying exposure and transmission processes, and for developing quantitative assessments and evaluations of alternative strategies needed to manage the pandemic [36]. Mechanistic modeling of epidemic dynamics originated with the publications of Ross [37] in 1910 and of Kermack and McKendrick in 1927 [38]; their work established a discipline that, as it evolved over time, has utilized a wide spectrum of methods, including Ordinary Differential Equations (ODEs), Difference Equations (DEs), Partial Differential Equations (PDEs), Integrodifferential Equations (IDEs), Cellular Automata (CA), Agent-Based Models (ABMs), Network Models, etc. (see, e.g., [39,40,41,42]). Many of these methods have been applied to studies of COVID-19; CDC [43] has been reporting predictions of COVID-19 spread and outcomes from an ensemble of over 30 publicly available models, which can be broadly grouped into three main categories, i.e., compartmental models, individual and network-based models, and statistical and machine learning models. Classic compartmental models, such as the SEIR (Susceptible, Exposed, Infectious, Recovered Individuals) model, represent a standard and widely used method in infectious disease epidemiology [44,45]. A SEIR model employs systems of either deterministic or stochastic ODEs to describe the dynamics of an epidemic. The pathogen transmission process is formulated as population transition/movement between “states” or “compartments”, comprising a dynamic system that is driven by epidemiological, biological, environmental and other related parameters at the community level. Due to their mechanism-based nature, compartmental models provide flexible frameworks for capturing epidemic dynamics, detecting potential resurgences, and exploring the efficacy of mitigation measures. Individual and network-based models, particularly agent-based models, expand on compartmental SEIR models by attempting to capture the complex interactions and behavior patterns of agents representing individuals in the populations of concern [46,47]. This approach can provide a more detailed description of disease transmission via a bottom-up framework, and potentially account for population heterogeneities that emulate “emergent” phenomena such as superspreading and spread within clusters. However, a reliable agent-based model generally poses excessive requirements for data inputs and parameters that need to be informative at local scales. Statistical and machine learning models use data-driven techniques aiming to predict future conditions by fitting curves defined through specified functions using early data [48], by capturing historical trends and correlations of time series [49], and by “learning” complex patterns and relationships between adverse outcomes and various underlying factors [50]. Statistical learning is valuable for modeling short-term trajectories of an epidemic; however, this approach alone cannot produce robust and reliable COVID-19 predictions without the support of mechanistic models, such as SEIR approaches. Statistical modeling has limited “extrapolation” ability (i.e., long-term prediction performance) due to its data-driven foundations and evaluations are typically conducted only for a few days ahead. The limitations of statistical modeling are especially true when attempting to describe COVID-19 spread and outcomes, since the data used to build the statistical model reflect conditions that are drastically changing temporally (due to changes in people’s behavior and responses, availability of treatments, possible virus adaptations, etc.) further curtailing its predictive ability. In contrast, mechanistic models can narrow the modeling/searching space by utilizing epidemiological knowledge to improve prediction performance [51].

As mentioned earlier, in the context of SEIR formulations, epidemic modeling frameworks can be either deterministic or stochastic [39]. The substantial uncertainties associated with the processes affecting COVID-19 dynamics, especially during the initial phases of the pandemic, severely limit the applicability of deterministic frameworks to “real world” studies of the pandemic. Stochastic frameworks are more appropriate for such studies as they can account for the inherent randomness and variability of the system and can generate the confidence intervals for each state variable via repeated simulations, starting with an estimated set of parameters and initial conditions. Monte Carlo approaches are then employed to consider the uncertainties of different model parameters within “most probable” domains. Instead of assuming homogeneous uniform mixing—as is the case with deterministic modeling (typically resulting in a system of ODEs)—stochastic frameworks can directly describe transmission heterogeneities via a hierarchical structure of uncertain parameters and simulate representative fat-tailed distributions that involve superspreader events [52]. Technical analyses of the relationships between the two frameworks can be found in the literature [53,54,55,56].

The majority of models discussed above have been predicting COVID-19 trajectories for countries or large areas within countries or states [57,58,59,60,61], an approach that does not account for important heterogeneities within these large geographical areas. Kain et al. [62] used a stochastic SEIR model to capture the heterogeneities of disease transmission from five selected counties/cities across the US and concluded that interventions truncating the individual-level transmission rate distribution while partially relaxing social distancing can be effective in maintaining epidemic control. Thomas et al. [63] used a network-based model to demonstrate that heterogeneity in population distribution can have large impacts on local pandemic timing and severity, based on simulations for 19 US cities. It should be mentioned that, although PDE-based approaches were not included in the CDC ensemble of models [43], they provide systematic ways to account for the effects of spatial heterogeneities on epidemic dynamics, and such approaches have been utilized in studies of the spread of COVID-19 in Arizona, US [64] and the Lombardy Region of Italy [65,66,67,68]. However, there has been a lack of modeling work focusing on evaluating the effectiveness of interventions that reduce exposures at local regions, while also exploring how key variables (e.g., population density, compliance with wearing face masks, prior exposures to various stressors, age stratification, comorbidities in the population, etc.) could affect disease dynamics and contribute to local heterogeneities of COVID-19 spread and outcomes. For example, the higher population density of urban settings causes social distancing to be more difficult to achieve than in suburban or rural areas and as a result populations of highly urbanized counties are expected to experience greater potential exposures, consequently resulting in higher numbers of cases and deaths. The deaths, however, are also related to the availability and quality of health care and hospitals, and these may be better in urban than in rural settings.

### 1.3. New Jersey as a “Microcosm” of COVID-19 Spread Heterogeneities

New Jersey has been one of the earliest and largest hotspots for COVID-19 in the United States, ranking first in the nation in per capita death rates until September 2021 (with over 300 deaths per 100,000 residents [69]) when it was surpassed by Mississippi. As mentioned earlier, in addition to individual-level risk factors, such as age, sex and underlying medical conditions, it has been widely recognized that multiple demographic, environmental and socioeconomic factors are strongly correlated with the patterns and severity of COVID-19 spread. In particular, racial and ethnic minorities, economically disadvantaged populations, and environmental justice communities have been disproportionally impacted by COVID-19. For example, over 47% of individuals with confirmed positive tests in NJ during the first wave of the pandemic were Black and Hispanic, while these minorities constitute approximately 31% of the State’s population; furthermore, the NJ counties with the highest rates of COVID-19 deaths (Essex, Union, Passaic, Hudson, Bergen) have historically high levels of hazardous air pollutants, such as airborne diesel exhaust particles, with concentrations that rank above the 98th percentile of national values. In fact, New Jersey is the State with the highest population density in the nation, with approximately 1208 people/mile^2^, while population densities of individual counties range from less than 200 people/mile^2^ (Salem County) to more than 14,600 people/mile^2^ (Hudson County). New Jersey is also one of the most ethnically and socioeconomically diverse States; furthermore, urban centers, suburban sprawl, an aging industrial infrastructure, active agriculture, extensive preservation areas and a densely populated coast, all in close proximity to each other, constitute a remarkably heterogeneous environmental landscape (Figure S1). Therefore, New Jersey is a veritable “microcosm” that is representative of much of what is happening across the entire contiguous US. As such, understanding the factors impacting the dynamics of COVID-19 across New Jersey can provide valuable insight and quantitative information that is directly applicable to a wide range of locations across the United States. It should be noted that the majority of casualties in New Jersey are associated with the first wave of the pandemic (March to September 2020), before effective pharmaceutical interventions became available and while uncertainties were persisting regarding both the significance of different exposure pathways and the proper medical protocols for treating hospitalized cases. This situation posed significant challenges with respect to both predicting trajectories of the disease across different regions of the State and evaluating the relevance and efficacy of intervention strategies for reducing exposures. Meeting these challenges required a flexible modeling framework, combining mechanistic description of stochastic epidemic dynamics with computationally efficient data science methods for parameter optimization.

In this article we present the development and deployment of a stochastic SEIR modeling platform that was designed to characterize and quantitatively assess the dynamics of the first wave of the pandemic for each individual county across New Jersey. To better quantify the effects of layered exposure interventions that are affected by spatial heterogeneities, we used state-of-the-art statistical inference tools and intelligent selection algorithms in a Sequential Quasi-Monte Carlo (SQMC) framework to reliably capture epidemic dynamics [70,71,72,73]. Model predictions, as well as other local information, for counties and municipalities across New Jersey, were made available to the public since the early days of the pandemic, via an online dashboard (https://ccl-eohsi.shinyapps.io/covid19_dashboard/ (accessed on 6 October 2021)). The findings of this work can provide guidance to local agencies for planning for future epidemics of infectious diseases, as it supports evaluation of mitigation strategies that can minimize potential exposures while allowing certain essential communal and economic activities.

## 2. Materials and Methods

### 2.1. Stochastic SEIR Model

**Figure 1:**
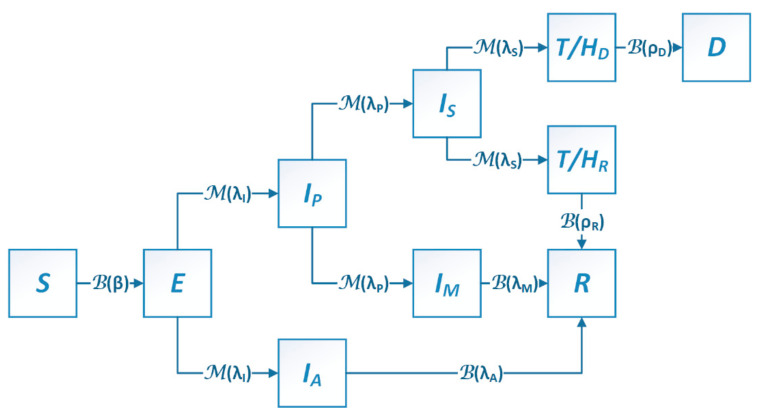
Block diagram of the stochastic compartmental SEIR model. Population “movements” between compartments (blocks representing stochastic states) follow binomial (B) or multinomial (M) distributions that are controlled by biological/epidemic parameters as marked on the arrows. *S* = susceptible, *E* = exposed, *I_p_* = presymptomatic, *I_A_* = asymptomatic, *I_s_* = severely symptomatic, *I_M_* = mildly symptomatic, *T/H_D_* = treated/hospitalized to death, *T/H_R_* = treated/hospitalized to recovery, *R* = recovered, *D* = dead. *β* = transmission rate. Definitions of other parameters are listed in Table S1.

A stochastic compartmental SEIR (Susceptible, Exposed, Infectious, Recovered Individuals) modeling system with time-varying probabilistic transmission parameters [45] was implemented in a Sequential Quasi-Monte Carlo framework to simulate local COVID-19 spread dynamics at the county level across New Jersey. The present model divides the residents of each county into ten compartments (Figure 1). Susceptible individuals (*S*) become exposed (*E*, infected but not yet infectious) through contacts with individuals from the infectious compartments comprised of presymptomatic, asymptomatic, and symptomatic individuals (*I_P_* + *I_A_* + *I_S_* + *I_M_*). Some of the exposed individuals become infectious but not yet symptomatic; these presymptomatic individuals (*I_p_*) become either mildly symptomatic (*I_M_*) or severely symptomatic (*I_S_*). For simplicity, it is assumed that individuals with mild symptoms can recover (*R*) without medical treatment and hospitalization, while those with severe symptoms should be treated/hospitalized (*T/H_D_* + *T/H_R_*). The treated/hospitalized individuals then progress to either death (*D*) or recovery (*R*) with a treatment/hospitalization fatality rate that can change over time. Because not all infected individuals show symptoms, some individuals progress to the asymptomatic compartment (*I_A_*) and to recovery (*R*) in the end. The above transmission dynamics constitute stochastic Markov Processes [74,75], where each compartment represents a discrete stochastic state. Transitions of individuals between compartments (equivalently, the stochastic states) are modeled probabilistically using binomial or multinomial distributions.

The mathematical relationships governing the dynamics of the stochastic SEIR model are listed in Equation (1): *C_A_*, *C_P_*, *C_M_* and *C_S_* denote the relative infectiousness of asymptomatic, presymptomatic, mildly symptomatic, and severely symptomatic individuals, respectively; *λ_I_*, *λ_P_*, *λ_A_*, *λ_S_* and *λ_M_* are rate constants representing the reciprocal of infectious periods for states *E*, *I_P_*, *I_A_*, *I_S_* and *I_M_*, respectively; *ρ_R_* and *ρ_D_* are rate constants representing the reciprocal of duration from treatment/hospitalization to either recovery or death, respectively; *α*, *μ* and *HFR* denote the proportion of asymptomatic infections, symptomatic infections that do not require treatment/hospitalization and treatment/hospitalization fatality, respectively, where *HFR* is assumed to have a linear decreasing trend for the first wave, satisfying *HFR* = *HFR*_0_ − *k∙t*. The time-varying transmission rate is denoted as *β_t_* = *β*_0_∙*γ_t_*, where *β*_0_ is a constant representing the basic transmission rate, and *γ_t_* is the exposure control-based intervention scaling factor at time *t*. The binomial and multinomial distributions are denoted as ℬ(∙) and ℳ(∙), respectively. The differential operator is “d”, so for example, d*SE* denotes the number of individuals transitioning from the Susceptible compartment/state to the Exposed compartment/state per time unit.

(1) $$\begin{array}{l} dSE\sim B\left(S,1-\exp\left(-\beta_{t}\frac{C_{A}I_{A}+C_{P}I_{P}+C_{M}I_{M}+C_{S}I_{S}}{N}\right)\right)\\ \left(\begin{array}{c} dEE\\ dEI_{A}\\ dEI_{P} \end{array}\right)\sim M\left(E,\left(\begin{array}{c} \exp(-\lambda_{I}dt)\\ \left(1-\exp(-\lambda_{I}dt\right))\alpha \\ \left(1-\exp(-\lambda_{I}dt\right))\left(1-\alpha \right) \end{array}\right)\right)\\ dI_{A}R\sim B\left(I_{A},1-\exp(-\lambda_{A}dt)\right)\\ \left(\begin{array}{c} dI_{P}I_{P}\\ dI_{P}I_{M}\\ dI_{P}I_{S} \end{array}\right)\sim M\left(I_{P},\left(\begin{array}{c} \exp(-\lambda_{P}dt)\\ \left(1-\exp(-\lambda_{P}dt\right))\mu \\ \left(1-\exp(-\lambda_{P}dt\right))\left(1-\mu \right) \end{array}\right)\right)\\ dI_{M}R\sim B\left(I_{M},1-\exp(-\lambda_{M}dt)\right)\\ \left(\begin{array}{c} dI_{S}I_{S}\\ dI_{S}H_{R}\\ dI_{S}H_{D} \end{array}\right)\sim M\left(I_{S},\left(\begin{array}{c} \exp(-\lambda_{S}dt)\\ \left(1-\exp(-\lambda_{S}dt\right))HFR\\ \left(1-\exp(-\lambda_{S}dt\right))\left(1-HFR\right) \end{array}\right)\right)\\ dH_{R}R\sim B\left(H_{R},1-\exp(-\rho_{R}dt)\right)\\ dH_{D}D\sim B\left(H_{D},1-\exp(-\rho_{D}dt)\right)\\ \frac{dS}{dt}=-dSE\\ \frac{dE}{dt}=dSE-dEI_{A}-dEI_{P}\\ \frac{dI_{A}}{dt}=dEI_{A}-dI_{A}R\\ \frac{dI_{P}}{dt}=dEI_{P}-dI_{P}I_{M}-dI_{P}I_{S}\\ \frac{dI_{M}}{dt}=dI_{P}I_{M}-dI_{M}R\\ \frac{dI_{S}}{dt}=dI_{P}I_{S}-dI_{S}H_{R}-dI_{S}H_{D}\\ \frac{dH_{R}}{dt}=dI_{S}H_{R}-dH_{R}R\\ \frac{dH_{D}}{dt}=dI_{S}H_{D}-dH_{D}D\\ \frac{dR}{dt}=dI_{A}R+dI_{M}R+dH_{R}R\\ \frac{dD}{dt}=dH_{D}D \end{array}$$

The biological and epidemic-relevant parameters that determine the compartmental transition probability and disease transmission dynamics are provided in the Supporting Information. These parameters were specified with either point (Table S1) or range estimates (Table S2), to account for various uncertainties associated with the dynamics of the first wave of the COVID-19 pandemic. Exposure control-based intervention strategies and public health policies are important factors affecting the transmission parameters, forcing them to vary over time. During early March, 2020, telecommuting or work-from-home arrangements were suggested/implemented for all businesses and non-profits that could operate in such a mode in the State. Following a series of mitigation measures (such as closures of educational facilities and non-essential services), the Governor issued a directive on 21 March, that residents should stay at home. When the Centers for Disease Control and Prevention (CDC) recommended wearing face masks to limit the spread of COVID-19 in early April 2020, the New Jersey Governor’s office implemented a face masks directive starting on April 11. In the model, we considered all of the above mitigation measures and policies. We divided the timeline into four intervention periods, i.e., non-intervention, work from home, shelter-in-place, and implementation of face masks directive, for estimating the effects of those measures on the spread of COVID-19 (Figure S4).

The treatment/hospitalization fatality rate (T/HFR) is an important metric for evaluating disease severity of COVID-19. A large T/HFR (e.g., 5–28%) was associated with the initial rise in the epidemic. After the initial period, as improved medical care protocols started becoming available, the T/HFR decreased [76]. Figure S5 depicts an approximate estimate of T/HFR in New Jersey, that decreased initially and then remained within a stable range. To account for the changing T/HFR that can affect COVID-19 death trajectories, we assumed a time-varying T/HFR beginning in mid-April (uncertain interval: 4/11–4/15). Note that April 14 is the date when the hospitalizations reached a peak; as such, we used a linear decreasing function, setting 1% as the lower bound of the estimated T/HFRs, to characterize such reduction and embedded it in the stochastic SEIR model.

### 2.2. Model Calibration

The stochastic SEIR model was calibrated using the reported daily numbers of new confirmed COVID-19 deaths for each NJ county, a metric that is substantially more reliable than other available data, such as the daily reported cases. The death data were based on reports from the New Jersey Department of Health (NJDOH) [77] and were compared with numbers reported in national repositories (NY Times, Atlantic’s COVID Tracking Project, Johns Hopkins). It should be noted that in late June 2020, New Jersey started reporting “probable” cases and deaths. A probable COVID-19 case/death is defined based on evidence from clinical, epidemiological or serological testing, or from vital records, but without a confirmatory laboratory RNA test. We excluded the probable deaths in implementing the model simulations in order to avoid discontinuities and fluctuations in the data and reduce related uncertainties. We adopted a SQMC framework for the optimization of the stochastic dynamic SEIR models, as such frameworks have been demonstrated to be more accurate and robust than other state/parameter estimation methods, especially for nonlinear and non-Gaussian dynamic systems [78]. The SQMC framework takes advantage of advanced filtering and sampling techniques (e.g., Bayesian Filtering and Sequential Monte Carlo) to “predict-and-correct” states recursively, in a manner consistent with the known dynamic model structure and the available observed data. In this study, an improved filtering algorithm [79], called Iterated Filtering [75], was used to optimize four parameters of the stochastic SEIR model, specifically a basic transmission rate ${\hat{\beta }}_{0}$, a shelter-in-place transmission scaling factor ${\hat{\gamma }}_{\mathrm{SIP}}$, a face masks directive transmission scaling factor ${\hat{\gamma }}_{\mathrm{FMD}}$, and a treatment/hospitalization fatality rate slope *k*_T/HFR_. The work from home transmission scaling factor $\gamma_{\mathrm{WFH}}$ was not estimated but was drawn randomly from a specified range (Table S2).

In order to consider the uncertainties associated with different model parameters, we generated 2000 parameter sets (low discrepancy Sobol sequences [45]) by “randomly” drawing samples from the specified parameter ranges. We constructed the model based on each Sobol sequence and calibrated the corresponding 2000 SEIR sub-models for each of the 21 counties of the State of New Jersey. The statistical inference and estimation algorithm has certain intrinsic limitations that in some cases may cause optimization failure (with non-convergent iteration), trapping into local optimum (with few initial values and particles), etc. To obtain reliable estimates for each calibration, we used 100 particle-filter iterations, 3000 particles and 5 particle filtering simulations [75]: these algorithm parameter settings ensure optimized performance while maintaining a practical computational load. We selected a time step of 4 h to ensure stable estimation for each state variable, and as such the daily predictions (number of deaths) are the sum of six estimates calculated in each day.

### 2.3. Simulation

The optimized parameters, along with the corresponding Sobol sequences, were combined to run multiple sub-simulations (2000) for each of the 21 NJ counties, where each sub-simulation was repeated multiple times (200) to account for stochasticity in state transitions. We considered three scenarios to assess the effects of mitigation measures on disease transmission: (a) *Baseline*, a counterfactual scenario using the estimated initial basic transmission rate ${\hat{\beta }}_{0}$ throughout the epidemic; (b) *Social Distancing without Face Masks*, a counterfactual scenario using the estimated shelter-in-place transmission rate ${\hat{\beta }}_{0}\cdot {\hat{\gamma }}_{\mathrm{SIP}}$ after March 21; and (c) *Social Distancing with Face Masks*, a “real world” scenario using the estimated shelter-in-place transmission rate ${\hat{\beta }}_{0}\cdot {\hat{\gamma }}_{\mathrm{SIP}}$ from March 21 to mid-April, and using the face masks directive transmission rate ${\hat{\beta }}_{0}\cdot {\hat{\gamma }}_{\mathrm{FMD}}$ after mid-April. A time-varying treatment/hospitalization fatality rate T/HFR was also included for the latter two scenarios (Figure S4).

The predicted death trajectories are the result of aggregated sub-simulations performed using the sets of different calibrated models with corresponding Sobol sequences. It should be noted that not all sub-simulations are suitable for evaluation. The reasons are twofold: (a) using relatively wide parameter ranges causes some Sobol sequences to deviate substantially from the underlying true parameter values. Accordingly, the optimized parameters based on these “biased” Sobol sequences, as well as the “biased” sub-models, do not reflect the exact epidemic dynamics; (b) due to the intrinsic limitations of the optimization algorithm, the estimated parameters from some sub-models do not represent “global” optima. These “poor” estimates tend to fail to capture the exact epidemic dynamics, so the need arises to systematically select the “proper” Sobol sequences. Kain et al. [62] selected Sobol sequences using a likelihood metric; however, that metric cannot adequately reflect the performance of the calibration process and may become invalid or even misleading, because it is calculated based on certain ideal distributions. For this reason, we designed an efficient algorithm for selecting the “most suitable” Sobol sequences and associated sub-models: we first select the “qualified” Sobol sequences that correspond to good predictions of both the daily new deaths and the total deaths (e.g., below an error tolerance with 30% of the maximum); we then use expert knowledge and screening rules (if necessary) to target the “most suitable” Sobol sequences (~10) located within the most frequent/stable intervals (Figure S6). The intensive computation for optimization and exploration of the global optimum, as well as the efficient algorithm used to select the “most suitable” Sobol sequences are crucial for capturing robust and reliable epidemic dynamics across different areas.

## 3. Results

**Figure 2:**
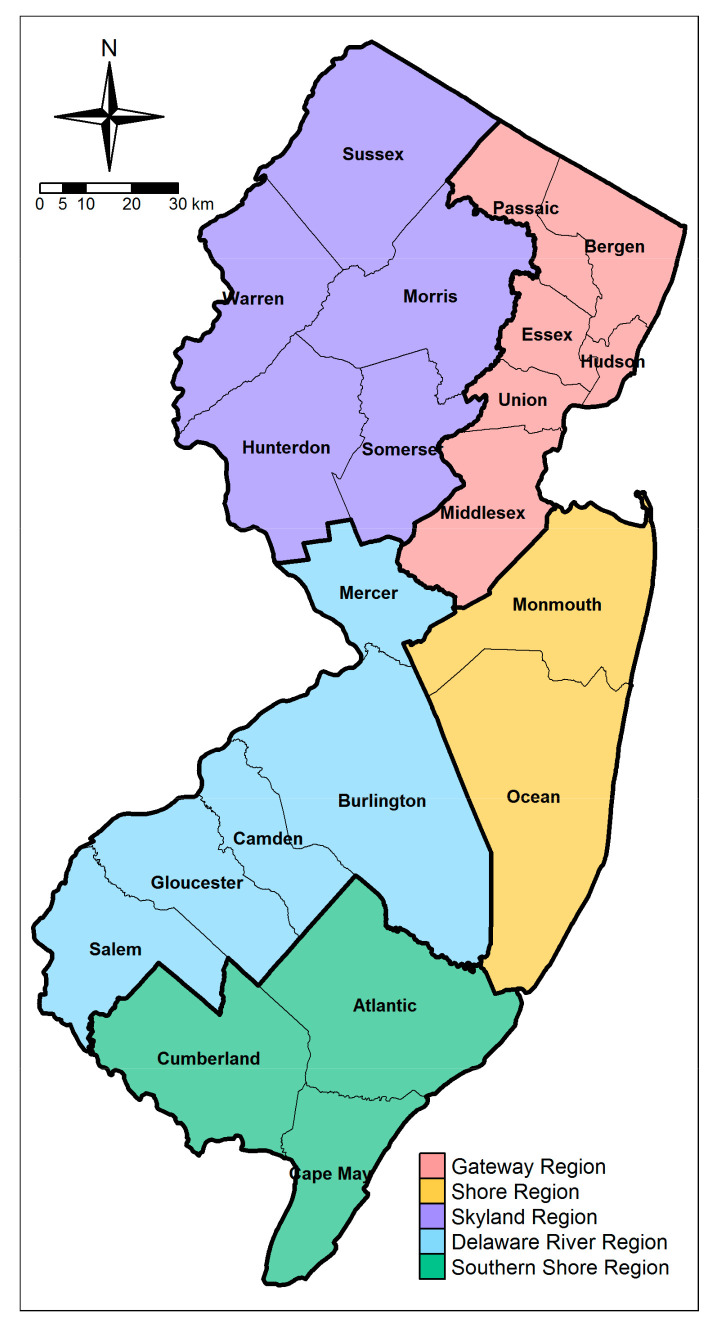
The 21 Counties and the five geographical regions identified based on similarities of exposure-relevant factors across the State of New Jersey.

The spatial domain considered in this study covers the entire State of New Jersey (with 21 counties), where five clustered geographic regions were identified to better explore the spatial heterogeneities of disease transmission (Figure 2).

### 3.1. Spatiotemporal Analysis of COVID-19 Deaths across New Jersey

**Figure 3:**
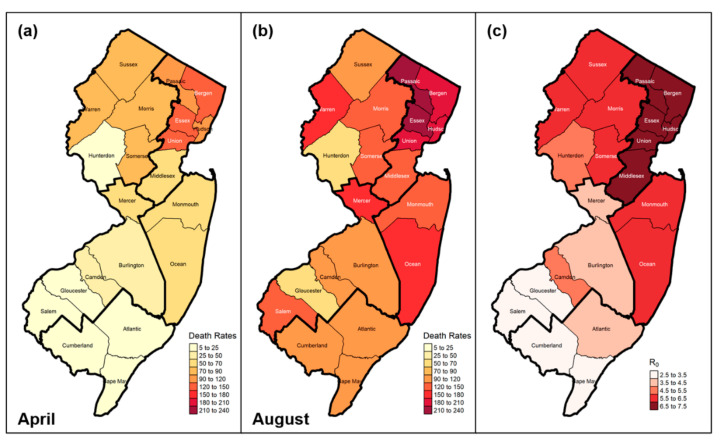
Spread of the COVID-19 epidemic across New Jersey: (**a**) death rates (confirmed deaths per 100,000 people) by the end of April 2020, (**b**) death rates by the end of August 2020, and (**c**) basic reproduction number (R_0_) at the county level.

Table 1 provides a summary overview of the COVID-19 death rates during the first wave of the pandemic for each of the 21 New Jersey counties, as well as for the five geographic regions identified in the map of Figure 2. The death rate is defined as the number of the confirmed deaths per 100,000 residents during a specified time period. The peaks occurred mainly in mid-April 2020 for areas near the epicenter of New York City (e.g., Gateway Region), while there was a two to four weeks delay for those in Southern New Jersey (e.g., Delaware River Region, Southern Shore Region). Until August 2020, areas in southern New Jersey had death rates ~40% lower than those in the north. Figure 3a,b show the spatial distribution of the death rates calculated for April and August 2020, respectively. The local variations of the death rates in April 2020 are significantly associated with the proximity to the “epicenter”, that is, the closer a county is to New York City (NYC), the higher was the death rate. However, this pattern became more complex by August 2020: for instance, counties far from the NYC “epicenter” may have larger death rates than those counties that are nearer (e.g., Salem and Gloucester in Figure 3a,b), indicating that the effects from other factors, such as population density, policy compliance, etc., became more prominent as the COVID-19 spread progressed.

### 3.2. Comparison of Predicted and Reported Confirmed Deaths

**Figure 4:**
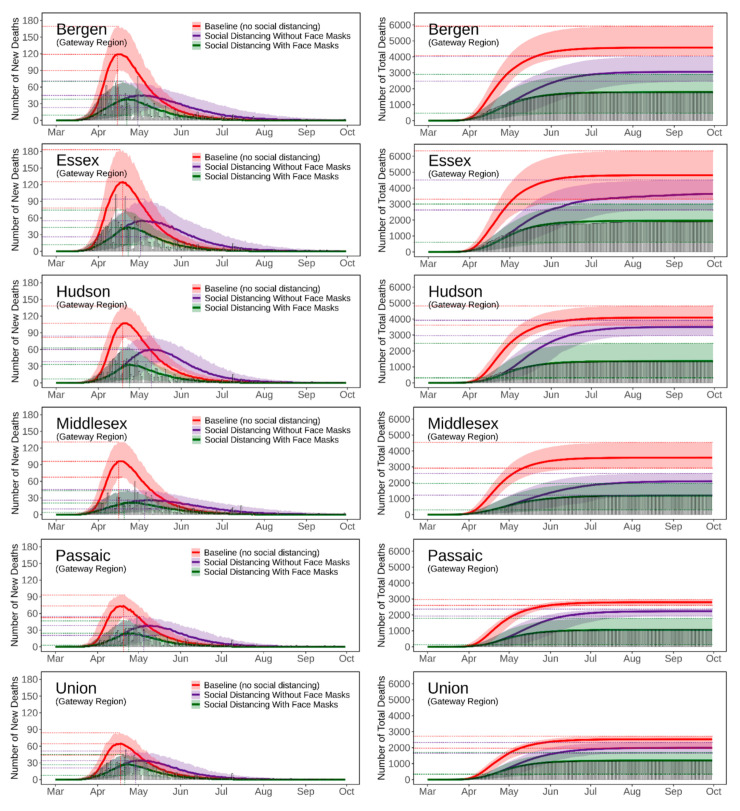
Predicted trajectories of new/total confirmed deaths from COVID-19 corresponding to three exposure scenarios for the counties in the Gateway Region of NJ, March to September 2020, compared with reported death data (vertical bars). The color-shaded areas depict the 95% Confidence Interval (CI) of the corresponding predictions.

**Figure 5:**
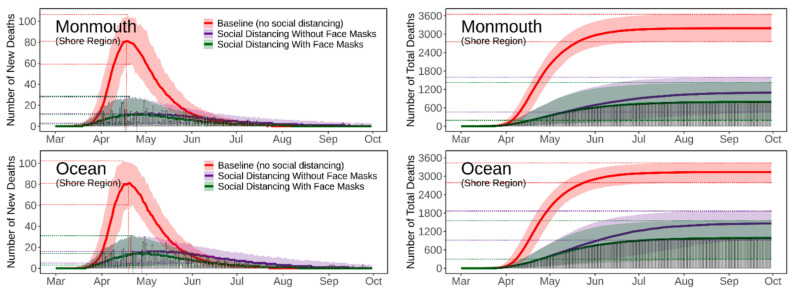
Predicted trajectories of new/total confirmed deaths from COVID-19 corresponding to three exposure scenarios for the counties in the Shore Region of NJ, March to September 2020, compared with reported death data (vertical bars). The color-shaded areas depict the 95% Confidence Interval (CI) of the corresponding predictions.

**Figure 6:**
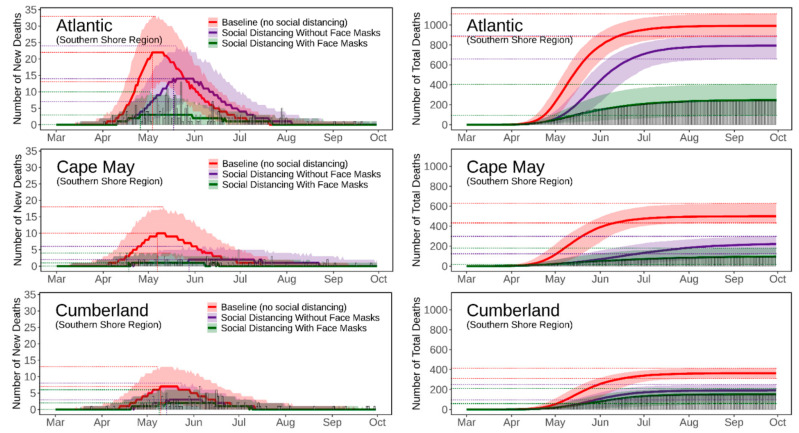
Predicted trajectories of new/total confirmed deaths from COVID-19 corresponding to three exposure scenarios for the counties in the Southern Shore Region of NJ, March to September 2020, compared with reported death data (vertical bars). The color-shaded areas depict the 95% Confidence Interval (CI) of the corresponding predictions.

**Figure 7:**
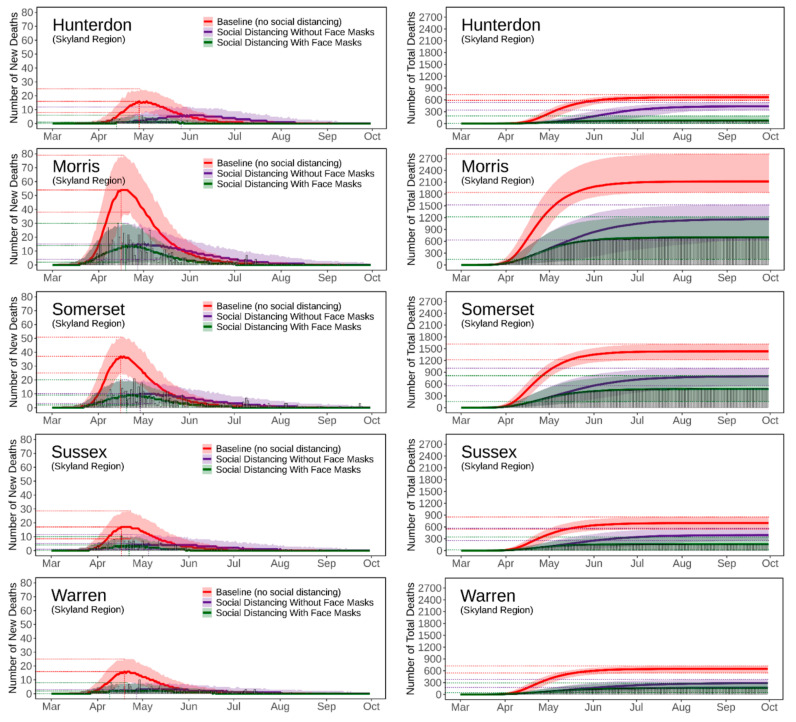
Predicted trajectories of new/total confirmed deaths from COVID-19 corresponding to three exposure scenarios for the counties in the Skyland Region of NJ, March to September 2020, compared with reported death data (vertical bars). The color-shaded areas depict the 95% Confidence Interval (CI) of the corresponding predictions.

**Figure 8:**
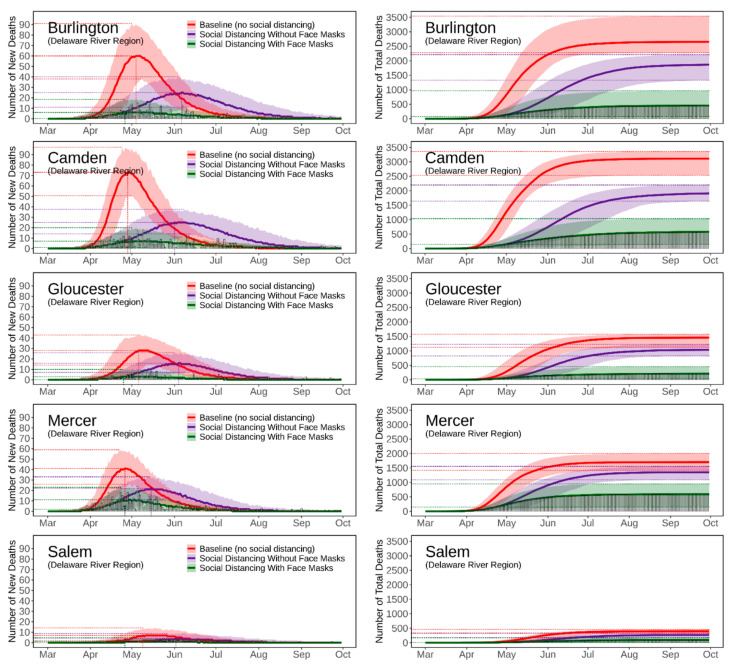
Predicted trajectories of new/total confirmed deaths from COVID-19 corresponding to three exposure scenarios for the counties in the Delaware River Region of NJ, March to September 2020, compared with reported death data (vertical bars). The color-shaded areas depict the 95% Confidence Interval (CI) of the corresponding predictions.

Figure 4, Figure 5, Figure 6, Figure 7 and Figure 8 present the predicted trajectories of the daily new/total confirmed deaths (with median and 95% confidence interval) for the three scenarios considered, along with the reported numbers of the confirmed deaths for each county. The same scale was used for the plots for counties within the same geographic region, in order to better show between/within-region variations. Models were calibrated with the daily new confirmed deaths before 2 August 2020 and were then tested for 8 days and 52 days ahead, i.e., 3 August to 10 August to 30 September. As mentioned earlier, regularly updated model predictions were made available to the public throughout the first wave of the pandemic via the dashboard: https://ccl-eohsi.shinyapps.io/covid19_dashboard/ (accessed on 6 October 2021).

Three statistical measures, i.e., root mean squared error (RMSE), mean absolute percentage error (MEPE), and envelope bias (EB), were used to evaluate model performance (Table S3). There is very good agreement between the predicted and reported numbers of total deaths. The reported total confirmed deaths are all within the 95% confidence interval for each county (except for Mercer with EB = 1 day), with MAPE ranging from 9% to 24%. The reported daily new deaths show inter-day fluctuations that, as expected, cannot be captured by the averaged estimates of the model, but most of which are within the 95% confidence interval. The RMSE of the daily new deaths varies for different regions, with the highest in the Gateway Region (larger cardinal death number); the RMSE in the rising phase of the first wave of the epidemic was generally lower than that in the falling phase.

### 3.3. Realistic and Counterfactual Intervention Scenarios

Figure S7 shows the predicted median of the new/total confirmed deaths from the 21 New Jersey counties for each of the three scenarios, i.e., *Baseline* (scenario 1), *Social Distancing without Face Masks* (scenario 2), *and Social Distancing with Face Masks* (scenario 3). When considering the situation of practicing social distancing but without wearing face masks (scenario 2 vs. scenario 1), the model predicts that there is a 16 [IQR (14, 21)] days delay of peaks, with 179 [IQR (115, 212)] deaths averted per 100,000 people. The effectiveness of social distancing in “flattening the curve” results from the significant reduction of disease transmission/exposure, according to the estimates of transmission rates shown in Figure S8. Adopting the face masks recommendation (scenario 3 vs. scenario 2), is shown to have saved an additional 169 [IQR (116, 281)] lives per 100,000 people compared to just using social distancing, and to have resulted in a peak delay of three [IQR (−2, 5)] days compared to the baseline scenario. The concurrent presence of social distancing and wearing face masks produce synergistic effects that considerably reduce deaths while slightly delaying the peaks; see Table S4 for the evaluation of effects for each NJ county.

## 4. Discussion

### 4.1. Spatially Heterogeneous Transmission Rate

**Figure 9:**
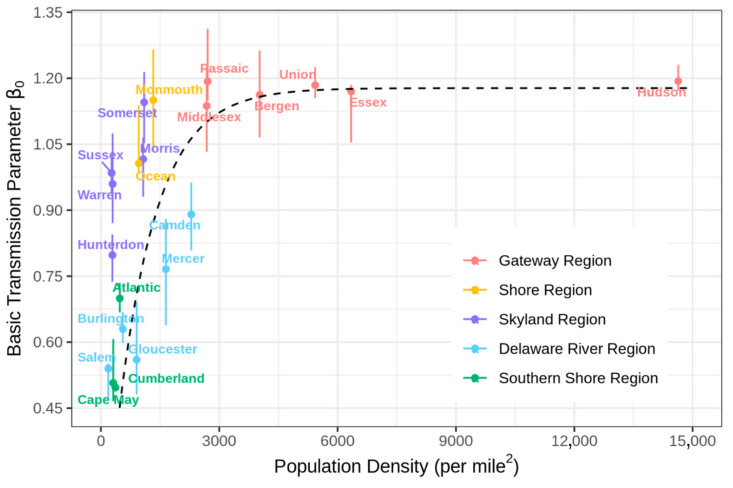
Nonlinear relationship between the estimated county level transmission rates and county population density. The black dashed line is a fitted mechanistic nonlinear scaling function consistent with kinetic theory modeling as described in [82].

The basic transmission rate (*β*_0_), a measure of the contacts per unit time (contact rate) multiplied by the likelihood of infection per contact, is a key component of the force of infection. Sy et al. [80] reported positive correlations between population density and the rates of transmission of SARS-CoV-2. However, this study was based on a statistical analysis (employing a linear mixed model) that cannot reveal the underlying mechanistic patterns. Our estimates for the transmission rates, optimized with the county-level SEIR models, reveal a nonlinear heterogeneous relationship to the population density (Figure 9). The basic transmission rate initially increases at lower population densities and then becomes saturated at higher densities (>2000 people/mile^2^). Such patterns reflect the combined effects of “density-dependent” (low density) and “frequency-dependent” (high density) mechanisms between contact rate and population density [81]. It should be pointed out that this trend is consistent with a mechanistic nonlinear scaling function (dashed line in Figure 9) that was derived from a spatial contact model which consider contacts of individuals within a population and is analogous to models that are widely used in kinetic theory [82]. Our estimation for the county-level transmission rates provides evidence on the significance of distinguishing density-dependent and frequency-dependent conditions, for better assessing the spatiotemporal patterns of the COVID-19 dynamic with partial differential equation models [83].

**Figure 10:**
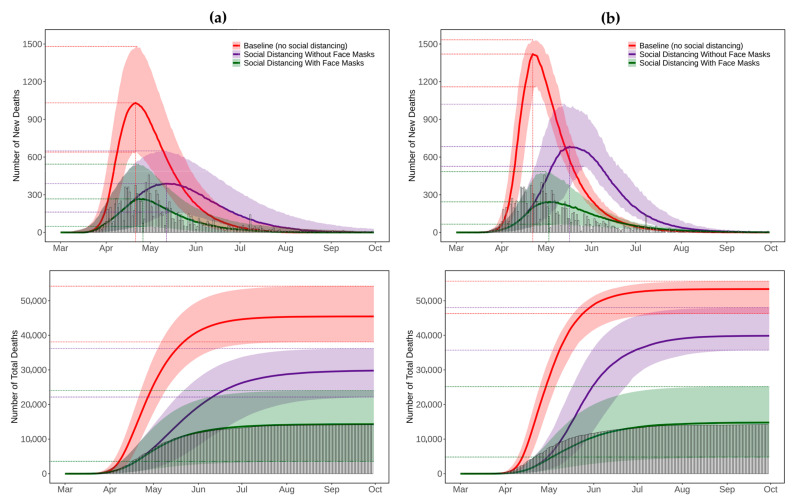
Comparison of projected trajectories of new/total deaths from COVID-19 calculated for different exposure scenarios with reported death data (vertical bars) for New Jersey using (**a**) summation of predictions from 21 county-level SEIR models, and (**b**) predictions from the statewide SEIR model. The color-shaded areas depict the 95% Confidence Interval (CI) of the corresponding predictions.

The transmission rate is also related to geographic location. Larger transmission rates were observed in locations closer to the epicenter of New York City (Figure S8a), with the highest rate occurring in the Gateway Region (1 to 1.3) and the lowest in the Southern Shore Region (0.4 to 0.7). Susceptible individuals living closer to the epicenter probably had a larger number of contacts with infectious individuals, due to both proximity to NYC and the higher population density of counties comprising this region. In practice, transmission rates are affected by different demographic (age, type of employment, income, education, ethnicity/race, etc.) and sociobehavioral factors, and the resulting spatially varying patterns are one of main reasons causing local heterogeneities in the spread and outcomes of COVID-19. Modeling at higher resolutions provides more flexible model structures which allow for capturing these local variations, and, moreover, improving the prediction performance at multiple scales. Figure 10 shows that the statewide death trajectory produced by combining outcomes from the 21 county-level SEIR models, considering heterogeneous transmission rates, is more reliable than the trajectory produced by a State-level SEIR model with a homogeneous transmission rate across the State.

### 4.2. Reproduction Number at County Level

The basic reproduction number (R_0_) is a fundamental epidemiologic metric used to describe the contagiousness of an infectious agent in the absence of interventions [84]: It is defined as the average number of secondary cases arising from a typical primary case in a completely susceptible population, and calculated as the product of the basic transmission rate and the duration of contagiousness for an infected person. The R_0_ for the present stochastic SEIR model is calculated via Equation (2):

(2) $$R_{0}=\beta_{0}\cdot \left[\alpha \cdot C_{A}\cdot \frac{1}{\lambda_{A}}+\left(1-\alpha \right)\cdot \mu \cdot \left(\frac{1}{\lambda_{P}}+\frac{1}{\lambda_{M}}\right)+\left(1-\alpha \right)\cdot \left(1-\mu \right)\cdot \left(\frac{1}{\lambda_{P}}+\frac{1}{\lambda_{S}}\right)\right]$$

where the terms in the square bracket represent the approximated duration of contagiousness (to recovery) that “averages” the effects of asymptomatic, mildly symptomatic, and severely symptomatic transmission. Early studies [85,86,87] reported an average estimated R_0_ for COVID-19 that was over 3 in multiple countries/regions with some estimates close to 6. Our estimates show significant variation across New Jersey, with R_0_ ranging from 3 to 7 (Figure 3c). Such variation is related to the spatial heterogeneity of the estimated transmission rates across New Jersey, as discussed in the previous subsection, as well as to the local uncertainties of the duration of contagiousness that are reflected in the values of the biological/epidemic parameters of the model, such as the relative infectiousness of asymptomatic individuals, and the proportions of asymptomatic, severely symptomatic, etc. individuals within the population (Equation (2), Table S2). Caution should be taken when comparing with estimates from previous studies, since R_0_ can vary considerably for different countries/regions [88]. It should also be pointed out that R_0_ is a model-dependent parameter and a proper comparison of R_0_ values should be based on the same model structure and assumptions [84].

The effective reproduction number (R_eff_) is a time-varying epidemiologic metric, defined as the actual average number of secondary cases arising from a typical primary case in a population that may have individuals with immunity and may have intervention measures in place at a specific time point [89]. In the present study, we assumed that individuals in the recovered state/compartment can directly gain immunity. The R_eff_ for the present stochastic SEIR model is calculated through Equation (3):

(3) $$R_{\mathrm{eff}}=frac\_S\cdot \gamma \cdot R_{0}$$

where *frac_S* is the fraction of the susceptible in time *t*; *γ* is the transmission scaling factor either specified in Table S2 or optimized via the SQMC framework, according to the type of the exposure control-based intervention strategies; R_0_ is the basic reproduction number calculated in Equation (2). Previous studies mainly used statistical methods [90,91,92] to estimate R_eff_ in a fast/timely manner for evaluating the daily contagiousness of COVID-19. In this work, we used the stochastic SEIR model to calculate the R_eff_ as the product of R_0_, the fraction of the susceptible population, and the transmission scaling factor given the interventions at time *t* (Equation (3)). The trend of our estimated R_eff_ (green lines in Figure S9) is consistent with that reported by NJDOH [90]: our county estimates of R_eff_ in May and June 2020 range from 0.18 to 0.86, compared to the estimates (0.6–0.9) at the state level by NJDOH. Figure S9 presents the time-varying R_eff_ for each of the 21 New Jersey counties for the three scenarios considered. The R_eff_ values for scenario 1 (red lines) decrease in a fast and smooth manner due to the rapid reduction of the susceptible population, and then converge to a small value (below 0.1). In scenario 2 (purple lines) and scenario 3 (green lines), the R_eff_ values decrease according to the strengths of the mitigation measures and the proportion of the susceptible population in each county.

### 4.3. Reducing COVID-19 Fatality by Layered Exposure-Relevant Interventions

Layered interventions, via interruption of multiple transmission routes and pathways, are efficient in reducing exposures to SARS-CoV-2. To control the epidemic, New Jersey implemented different levels of social distancing since March 2020: keeping six feet from others can prevent transmission from direct droplet spray contact; school closures, mass gatherings bans, and shelter-in-place directives also directly reduce the contact rate. The recommendation to wear face masks in mid-April further reduced the likelihood of infection per contact, by limiting the transfer of viral droplets and aerosols [93]. In essence, the goal of the combined interventions was to scale (reduce) the transmission rates of COVID-19, as expressed in the SEIR model. The modeling results show that social distancing, particularly the shelter-in-place order, considerably reduced the county-level transmission rates from 0.92 [IQR (0.61, 1.07)] (Figure 9 and Figure S8a) to 0.30 [IQR (0.15, 0.55)] (33% [IQR (24%, 51%)] reduction) (Figure S8b), and that the face masks directive further reduced the transmission rates to 0.20 [IQR (0.10, 0.41)] (65% [IQR (50%, 75%)] reduction) (Figure S8c). It should be noted that “social” (physical) distancing involves compliance with a series of government directives and guidelines mentioned above, though shelter-in-place is expected to play a dominant role in reducing the transmission rate before the implementation of face masks directive.

Population density not only affects the (basic) transmission rate, as discussed in the previous subsection, but also affects the effectiveness of the interventions/policies that scale the transmission rate. According to the counterfactual analysis for scenario 2, i.e., *Social Distancing without Face Masks*, counties where more deaths per capita were averted by social distancing are those with lower population density (Figure S10a). This can be explained by the fact that social distancing is usually more difficult to achieve in densely populated areas. By comparing scenario 2 and scenario 3 (*Social Distancing with Face Masks*), it is concluded that the face masks directive was more effective in areas with higher population density, i.e., the difference in averted deaths per capita (Figure S10b) between scenario 3 and scenario 2 is more negative, and the corresponding ratio (Figure S10c) is larger than 1. This could indicate that residents of more densely populated areas tend to be more concerned and exhibit higher compliance [94] with wearing face masks. Another possible explanation is that when social distancing can be accomplished in low population density areas, the effectiveness of a mask is lower, since emitted droplets and aerosols do not commonly reach a large number of individuals. The other issue is how people travel to work: in dense cities, there is substantially higher use of mass transit where masks are required and are effective, while in rural areas private automobiles are more likely used and masks would not offer additional benefits.

In addition to the transmission rate, the fatality rate is another crucial metric that determines death trajectories. We modeled the decline of treatment/hospitalization fatality rate (T/HFR) when the hospitalizations reached their peak in New Jersey. The estimates for the decline slope of the T/HFR curve is −0.07% [IQR (−0.09%, −0.05%)], consistent with the estimate of −0.06% reported by CEBM [76]. Such a decline (presumably due to improved treatment protocols) saved 38 [IQR (28, 60)] lives per 100,000 people, a much lower number than the number of lives saved by layered interventions, i.e., 357 [IQR (290, 429)]. This indicates that layered interventions, aiming to mitigate exposures to SARS-CoV-2, have been more effective in reducing mortality than medical treatments available during the first wave of the pandemic.

### 4.4. Limitations

There are certain limitations to the study presented here. First, this version of the stochastic SEIR model does not account explicitly for heterogeneities due to age disparities in susceptibility and transmission patterns [95]; incorporating age stratification should further reveal patterns in transmission/fatality rates (Figure 9 and Figure S10) with respect to population density and relevant demographic factors. Though we have shown the benefits of prediction at finer spatial scales (Figure 10), in the present work, transmission of COVID-19 was modeled independently for each county without considering the effects of inter-county interactions; incorporating relevant information (such as population mobility) could have further improved the assessments of spatial transmission [64,83,96,97]; however, it should be recognized that the data-driven parameter estimation and optimization implicitly captures the effects of inter-county differences such as “differentials” in community levels. Second, the model calibration process considers key elements of transmission dynamics for different intervention measures and timing, while for simplicity, it assumes fixed transmission rates after the implementation of those mitigation measures. The current model was calibrated with the number of the reported confirmed deaths (with less fluctuation and uncertainty) and evaluated using the same type of the data. Numbers of projected new/total cases can be generated but would be expected to be much higher than numbers of reported cases. Using reported case data in model calibration to predict and assess case trajectories for each of the 21 NJ counties would have required considering additional parameters (such as COVID-19 testing rates) associated with great uncertainty and variability, particularly in the early phase of the pandemic. Comparisons and analyses of the predicted case trajectories were therefore not a main focus of this study. Third, it is known that a large proportion of deaths in New Jersey and across the nation particularly in the beginning of the epidemic, were associated with Long Term Care (LTC) facilities. Due to the unavailability of relevant data, when the model was implemented in the first months of the pandemic, an “average” fatality rate that did not distinguish deaths that occurred at hospitals from those that occurred at nursing home was used. Given that spread of COVID-19 in LTC facilities exhibited more rapid patterns, it could have been described separately from the spread occurring in populations surrounding around these facilities [77]. However, the stochastic SEIR framework that is presented here can actually model the effects of “superspreading” events, such as those that occurred in LTC facilities, by probabilistically considering individual-level heterogeneities in transmission as the stochastic SEIR model can capture “superspreader” events in the tails of the skewed distributions. It should be noted that the current model assumes a single average transmission rate which may lead to overestimation of effectiveness of some interventions in LTC facilities (e.g., social distancing is difficult to achieve in LTC due to residents’ frailty and close living quarters). However, the estimated population-average county transmission rates are significantly associated with the number of LTC beds (Pearson coefficient 0.68) in the corresponding counties: these parameters can provide crucial information for further optimization that would consider additional effects of heterogeneities within each county. Overall, COVID-19 spread is a very complex process that is affected by multiple demographic, socioeconomic/behavioral, and environmental factors. Tiered modeling can be useful in extracting complex mechanisms consistent with alternative hypotheses, while it should be complemented by statistical analysis involving large numbers of exposure-relevant factors [98], a task that is currently an on-going next step of this work [99]. Better understanding of transmission dynamics and characterization of critical parameters/factors can help guide individual and government decision making aiming to mitigate the future trajectory of any epidemic.

## 5. Conclusions

This article presents a hybrid application of systems science, employing a mechanistic stochastic SEIR modeling framework, combined with computational data science, using a Sequential Quasi-Monte Carlo framework for optimal estimation of multiple model parameters, for quantifying the dynamics of COVID-19 within each of the 21 counties of New Jersey. This work applied stochastic epidemiological modeling to multiple heterogeneous jurisdictions within a single region, where the same mitigation policies had been imposed, to investigate the effectiveness and compliance of layered exposure-relevant interventions (such as social distancing and face mask use) on epidemic control. The modeling results in fact revealed significant local heterogeneities of COVID-19 dynamics across New Jersey; the captured spatiotemporal patterns were found to be related to a wide range of factors, such as population density, proximity to the epicenter of the epidemic, and compliance with wearing face masks.

We estimated transmission rates varying both in space and time and derived a nonlinear relationship with respect to population density that is consistent with mechanistic predictions analogous to those derived in kinetic theory. Population density was also found to be related to the effectiveness and compliance of policies that “scale” the epidemic transmission rate. The modeling results indicate that social distancing and wearing face masks were effective in reducing exposures to SARS-CoV-2, averting an estimated 357 deaths [IQR (290, 429)] per 100,000 people while slightly delaying the peak by three [IQR (−2, 5)] days for the first wave in New Jersey. Furthermore, before effective pharmacological interventions became available, gradual improvements of treatment protocols during the first wave saved lives (38 [IQR (28, 60)] per 100,000 people) but at a much lower rate than of the lives saved by layered exposure interventions. The present computational modeling framework is currently being expanded to the study of socioeconomic, behavioral and environmental factors on pandemic dynamics [99] and to quantification and assessments of pharmaceutical interventions, such as vaccination and loss of immunity, that are currently of public concern [100,101,102].

## Figures and Tables

**Table 1:** COVID-19 confirmed deaths per 100,000 residents by August 31 2020 across New Jersey. The color bars in the time strip denote the weekly deaths per 100,000 people.

County/ State	Total Deaths	Deaths per 100,000	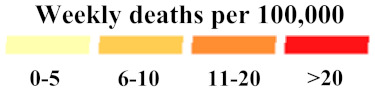	Geographic Region	Total Deaths	Deaths per 100,000	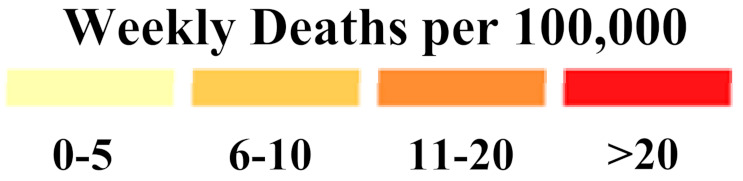
New Jersey	14,068	158	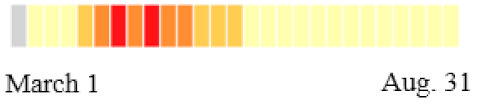				
Bergen	1768	189	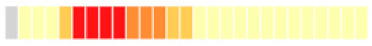	Gateway Region	8458	197	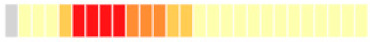
Essex	1868	234	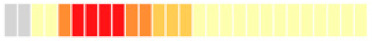				
Hudson	1336	198	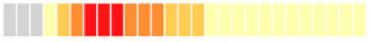				
Middlesex	1218	147	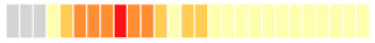				
Passaic	1098	218	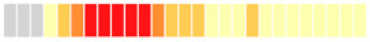				
Union	1170	210	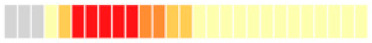				
Monmouth	763	123	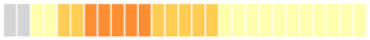	Shore Region	1715	140	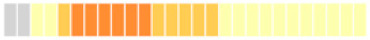
Ocean	952	158	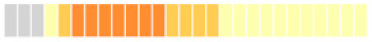				
Hunterdon	69	55	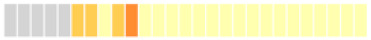	Skyland Region	1559	130	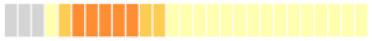
Morris	684	138	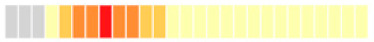				
Somerset	487	147	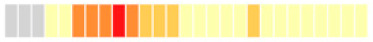				
Sussex	159	113	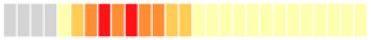				
Warren	160	151	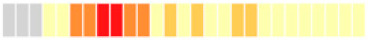				
Burlington	440	99	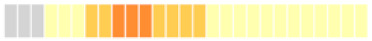	Delaware River Region	1866	111	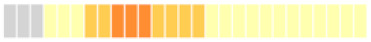
Camden	541	107	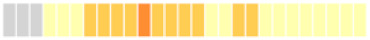				
Gloucester	211	72	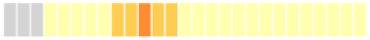				
Mercer	589	159	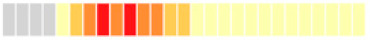				
Salem	85	136	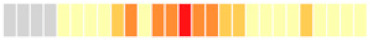				
Atlantic	237	89	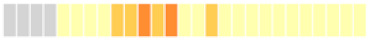	Southern Shore Region	470	92	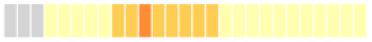
Cape May	90	97	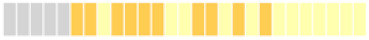				
Cumberland	143	95	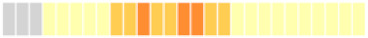				

## Data Availability

An online dashboard for this project is accessible at https://ccl-eohsi.shinyapps.io/covid19_dashboard/ (accessed on 6 October 2021). Data on COVID-19 cases and deaths are publicly available on the following URL: https://raw.githubusercontent.com/nytimes/covid-19-data/master/us-counties.csv (accessed on 6 October 2021).

## Supplementary Materials

The following are available online at https://www.mdpi.com/article/10.3390/ijerph182211950/s1, Figure S1: heterogeneity and diversity in demographic and environmental attributes across NJ; Figure S2: NJ resident deaths, Figure S3: numbers of new confirmed COVID-19 cases at municipality level in NJ from March to September 2020, Figure S4: schematic diagram of the stochastic SEIR model with time-varying transmission rate, Figure S5: approximated treatment/hospitalization fatality rate in NJ, Figure S6: illustration of the selection process for the estimated treatment/hospitalization fatality rates, Figure S7: projected median of new/total deaths from COVID-19 for three exposure scenarios. Figure S8: spatial distribution of the median estimates for basic transmission parameter and transmission scaling factors, Figure S9: time-varying effective reproduction number, Figure S10: effectiveness of social distancing and wearing face masks with respect to population density in five categories. Figure S11: screenshots of different tabs of the dashboard for the COVID-19 project. Table S1: Biological and epidemic-relevant parameters specified with point estimates for New Jersey, Table S2: biological and epidemic-relevant parameters specified with range estimates for NJ, Table S3: statistical measures of agreement between modeled and reported confirmed COVID-19 death data, Table S4: counterfactual analysis: deaths averted per 100,000 people and peak date delayed due to social distancing and face masks use.
