# Supplementary material for: Modeling Effects of Spatial Heterogeneities and Layered Exposure Interventions on the Spread of COVID-19 across New Jersey
Source: Int J Environ Res Public Health. 2021 Nov 14;18(22):11950. doi: 10.3390/ijerph182211950 (PMC8618648; doi:10.3390/ijerph182211950)

Table S1. Biological and epidemic-relevant parameters specified with point estimates for New Jersey.

Parameter	Value	Description
C_P, C_M, C_S	1	Relative infectiousness of presymptomatic, mildly symptomatic, and severely symptomatic
λ_I	1/4.5 days	1/Preinfectious period
λ_P	1/1.5 days	1/Presymptomatic infectious period
λ_A	1/7 days	1/Asymptomatic infectious period
λ_S	1/5.5 days	1/Severe infectious period until treatment/hospitalization
λ_M	1/5.5 days	1/Mild infectious period
ρ_R	1/20.5 days	1/Duration of treatment/hospitalization to recovery
ρ_D	1/17.8 days	1/Duration of treatment/hospitalization to death
HFR_{min}	0.01	Minimum treatment/hospitalization fatality rate (for the linear decreasing scenario)
SIP_{start}	2020-3-21	Shelter-in-place start date
Sim_{start}	2020-3-1	Simulation start date
Sim_{end}	2020-9-30	Simulation end date
Fit_{end}	2020-8-2	Latest date for data fitting
pop		Population size of the focal areas

Table S2. Biological and epidemic-relevant parameters specified with range estimates for New Jersey.

Parameter	Lower Bound	Upper Bound	Description
C_A	0.4	0.8	Relative infectiousness of asymptomatic
α	0.3	0.5	Proportion of asymptomatic infections
$1-\mu$	0.025	0.075	Proportion of symptomatic infections that require treatment/hospitalization
HFR_0	0.05	0.28	Initial treatment/hospitalization fatality rate
E_0	3	6	Number of initial infections
γ_{WFH}	0.7	0.95	Work from home transmission scaling factor
Epi_{start}	2020-1-15	2020-2-25	Start date of the epidemic
WFH_{start}	2020-3-6	2020-3-15	Work from home start date
EMM_{start}	2020-4-11	2020-4-15	Start date of enhanced mitigation measures

Table S3. Statistical measures of agreement between modeled and reported confirmed COVID-19 death data across the 21 New Jersey counties. RMSE = root mean squared error, MAPE = mean absolute percentage error, EB = envelope bias (number of days the reported data beyond the 95% confidence interval). The model evaluations are performed for a long period (March 1, 2020 - August 10, 2020) in a total of 163 days.

Geographic region	County	New deaths				Total deaths			
		RMSE	RMSE (rising)	RMSE (falling)	EB	MAPE	MAPE (rising)	MAPE (falling)	EB
Gateway Region	Bergen	10.8	15.48	6.88	23	0.10	0.24	0.02	0
	Essex	12.46	16.85	10.89	27	0.15	0.45	0.08	0
	Hudson	7.71	9.74	6.87	17	0.14	0.54	0.06	0
	Middlesex	6.96	8.97	5.79	18	0.24	0.84	0.02	0
	Passaic	5.82	7.83	4.42	18	0.09	0.26	0.02	0
	Union	5.93	7.25	5.31	10	0.17	0.69	0.02	0
Shore Region	Monmouth	3.66	4.46	3.17	7	0.13	0.40	0.01	0
	Ocean	4.29	4.97	4.02	6	0.13	0.54	0.02	0
Skyland Region	Hunterdon	0.90	1.22	0.72	2	0.10	0.34	0.04	0
	Morris	4.30	5.65	3.83	10	0.24	1.22	0.04	0
	Somerset	2.93	3.88	2.48	12	0.13	0.45	0.02	0
	Sussex	1.45	2.18	1.19	6	0.15	0.79	0.04	0
	Warren	1.44	2.05	1.09	3	0.24	0.75	0.06	0
Delaware River Region	Burlington	2.86	2.50	2.96	4	0.09	0.36	0.03	0
	Camden	2.97	3.32	2.69	5	0.21	0.57	0.03	0
	Gloucester	1.62	1.59	1.64	3	0.12	0.32	0.03	0
	Mercer	3.20	4.25	2.45	9	0.16	0.55	0.02	1
	Salem	0.81	0.85	0.74	1	0.16	0.23	0.05	0
Southern Shore Region	Atlantic	2.04	2.51	1.55	5	0.11	0.25	0.03	0
	Cape May	1.02	1.11	0.76	4	0.11	0.15	0.02	0
	Cumberland	1.27	1.29	1.25	2	0.15	0.28	0.02	0

Table S4. Counterfactual analysis: Deaths averted per 100,000 people and peak date delayed due to social distancing and face masks use across the 21 New Jersey counties.

Geographic region	County	Social distancing		Wearing face masks	
		Death rate averted	Peak date delayed	Death rate averted	Peak date delayed
Gateway Region	Bergen	173 (117, 218)	13 (8, 23)	131 (122, 203)	6 (3, 8)
	Essex	144 (69, 236)	16 (15, 18)	214 (115, 294)	5 (2, 6)
	Hudson	91 (76, 132)	21 (19, 24)	320 (265, 356)	3 (2, 9)
	Middlesex	177 (146, 276)	24 (10, 25)	116 (44, 138)	8 (4, 12)
	Passaic	115 (88, 129)	19 (14, 23)	232 (229, 233)	8 (2, 10)
	Union	80 (53, 108)	15 (11, 18)	141 (105, 184)	4 (4, 6)
Shore Region	Monmouth	336 (282, 395)	14 (11, 15)	47 (38, 60)	7 (3, 10)
	Ocean	282 (247, 301)	19 (7, 24)	84 (55, 99)	5 (2, 16)
Skyland Region	Hunterdon	179 (153, 213)	27 (19, 36)	295 (257, 333)	-14 (-18, 3)
	Morris	199 (172, 290)	13 (5, 24)	99 (49, 117)	6 (2, 8)
	Somerset	197 (144, 223)	17 (5, 21)	96 (78, 123)	2 (0, 7)
	Sussex	212 (174, 260)	14 (7, 22)	169 (122, 244)	0 (-3, 5)
	Warren	334 (279, 399)	14 (2, 29)	119 (75, 144)	2 (-12, 6)
Delaware River Region	Burlington	187 (170, 311)	29 (23, 38)	316 (231, 370)	-2 (-8, 8)
	Camden	217 (162, 249)	39 (33, 42)	281 (238, 293)	4 (1, 11)
	Gloucester	120 (95, 155)	20 (16, 31)	286 (236, 327)	-18 (-22, -7)
	Mercer	93 (76, 118)	22 (14, 23)	208 (168, 244)	4 (-3, 7)
	Salem	200 (176, 233)	16 (4, 29)	289 (198, 325)	-20 (-25, -17)
Southern Shore Region	Atlantic	74 (62, 96)	15 (11, 21)	208 (172, 223)	-2 (-11, 8)
	Cape May	296 (284, 371)	11 (4, 35)	133 (93, 188)	-19 (-27, -15)
	Cumberland	113 (99, 137)	3 (-9, 16)	26 (13, 41)	-3 (-10, 12)

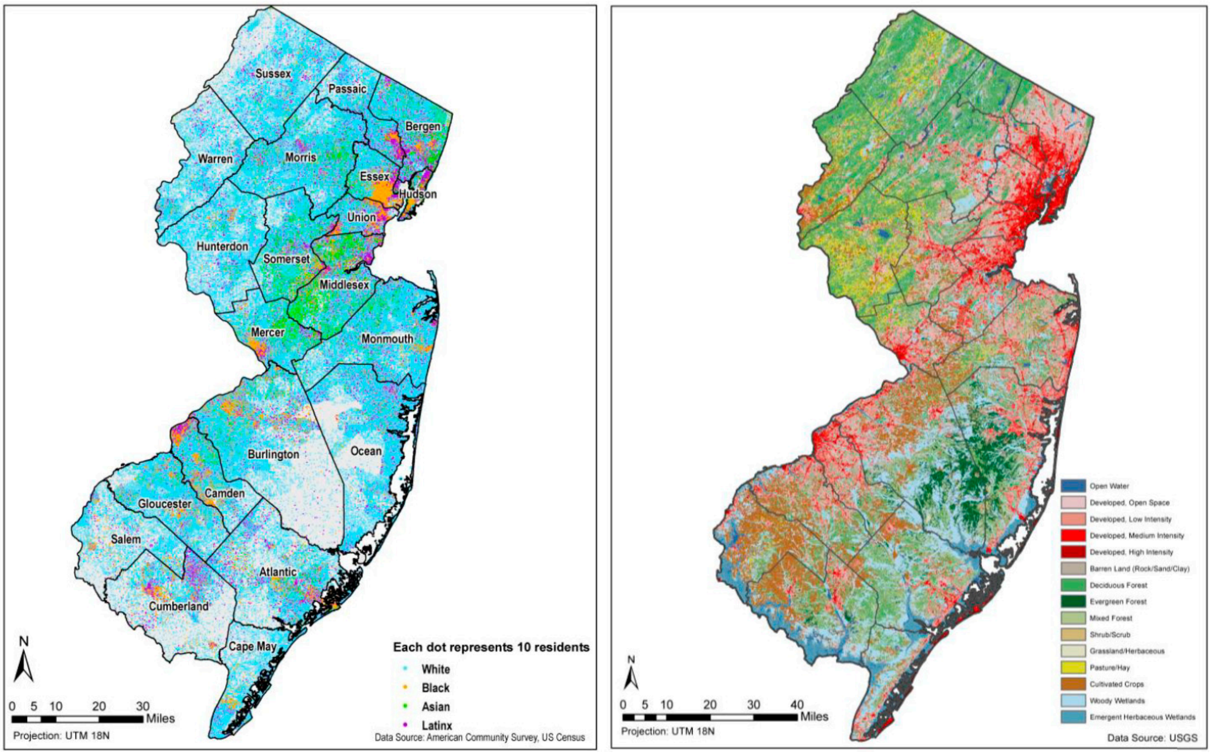

Figure S1. Heterogeneity and diversity in demographic (left) and environmental attributes (right) across New Jersey; the map on the left shows the ethnic diversity and the map on the right the heterogeneity in land use across the State.

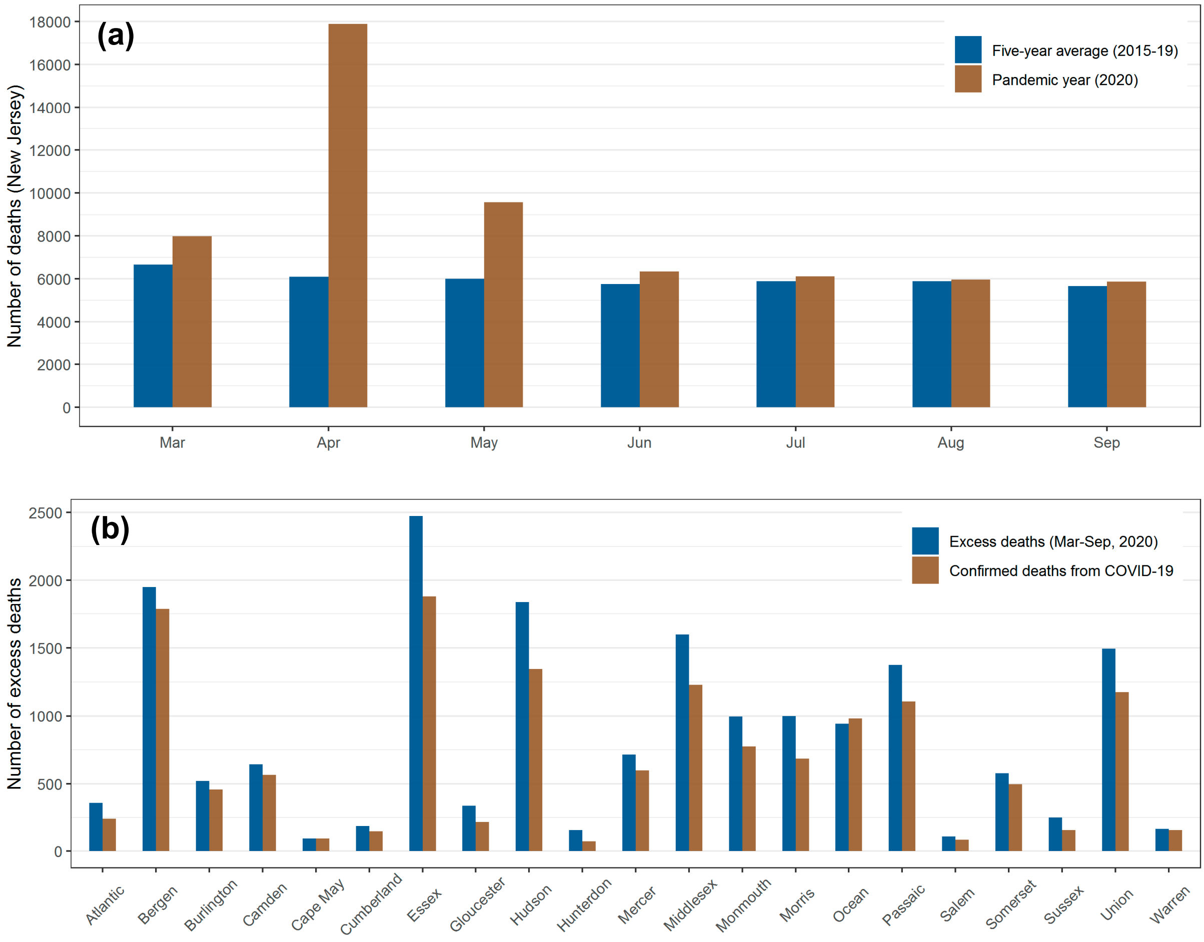

Figure S2. New Jersey resident deaths (data available at https://www.state.nj.us/health/chs/documents/2015-2020%20Deaths%20by%20Month%20and%20County%20of%20Residence.pdf): (a) the monthly deaths for five-year average (2015-2019) versus the resident deaths for the pandemic year (2020); (b) the county-level excess deaths (= resident deaths in 2020 - five-year average resident deaths) from March 1 to September 30 versus the reported confirmed COVID-19 deaths.

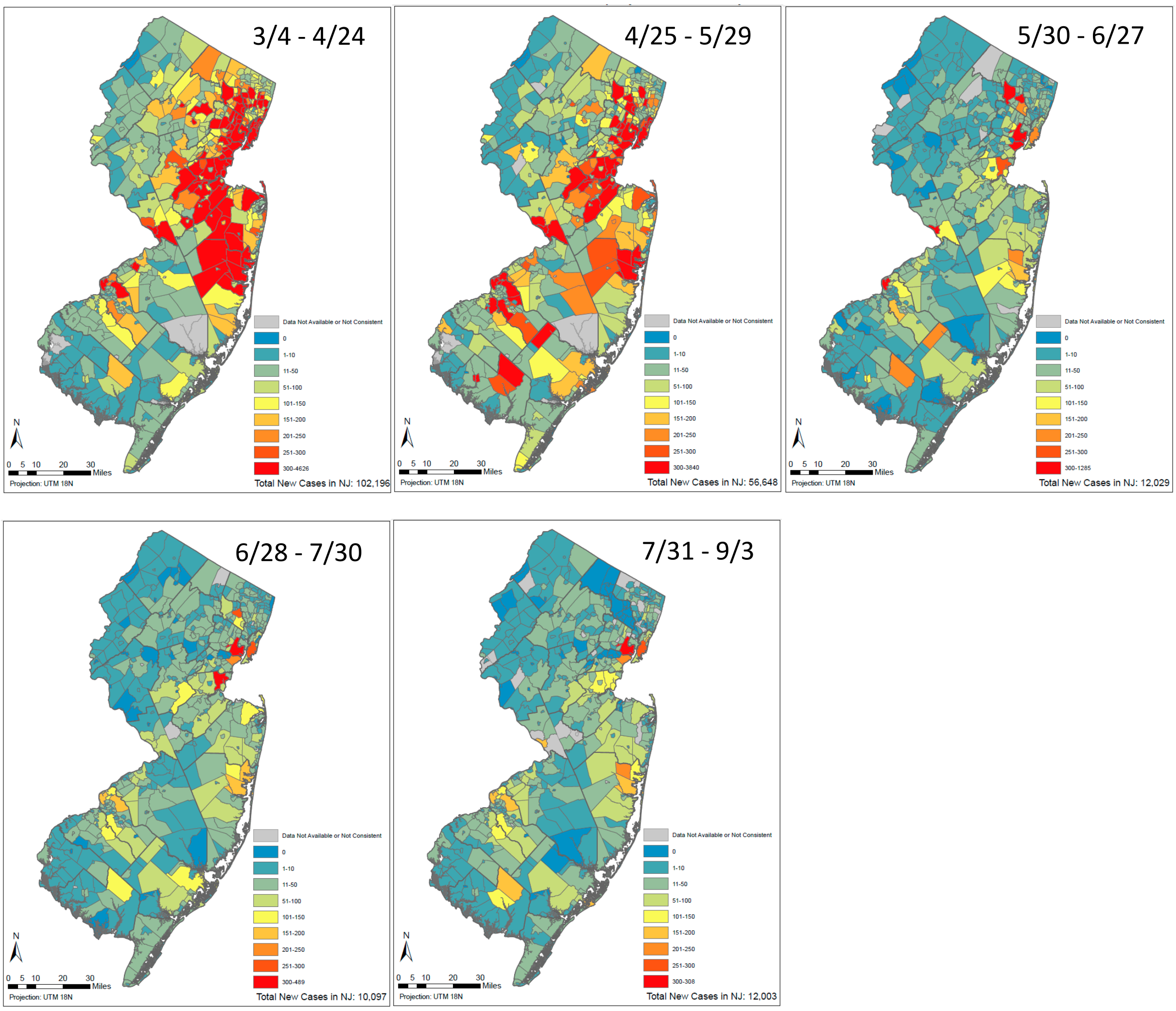

Figure S3. Numbers of new confirmed COVID-19 cases at municipality level in New Jersey from March to September, 2020; the maps demonstrate the heterogeneity of disease spread across the State.

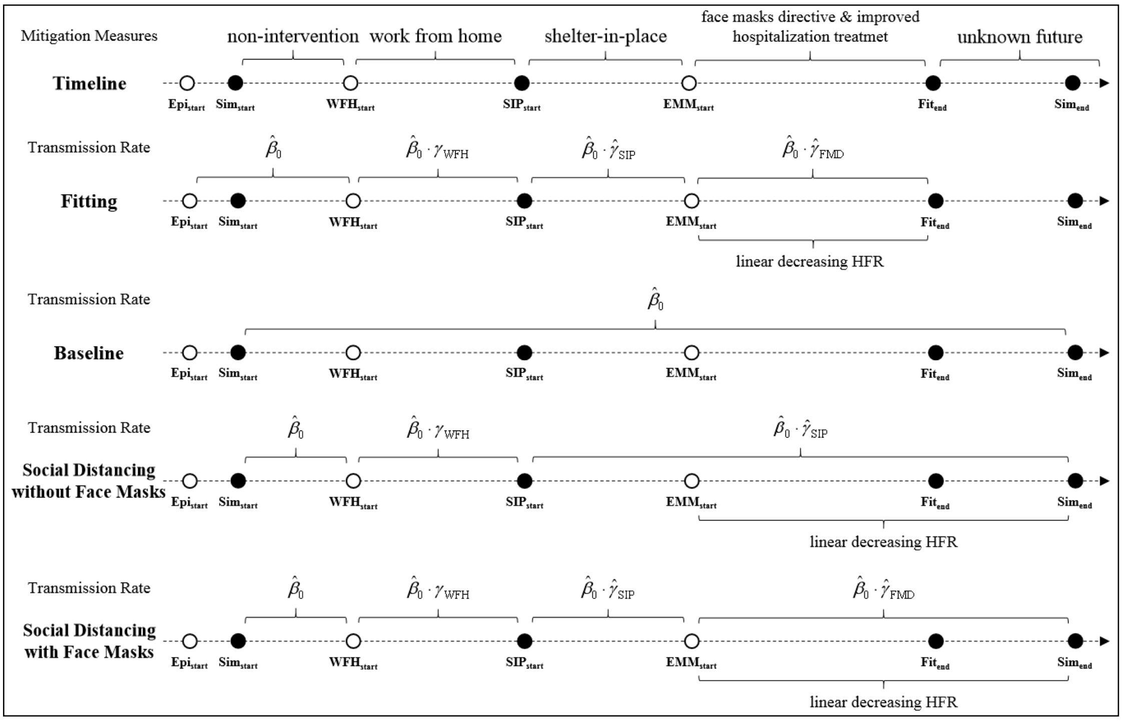

Figure S4. Schematic diagram of the stochastic SEIR model with time-varying transmission rate, showing the timeline of mitigation measures/policies implemented in New Jersey, fitting of models with typical intervention and timing, and the simulations for three scenarios (Baseline, Social Distancing without Face Masks, Social Distancing with Face Masks). The solid circles denote the time specified with point estimates (Table S1), and the hollow circles denote the time specified with range estimates (Table S2).

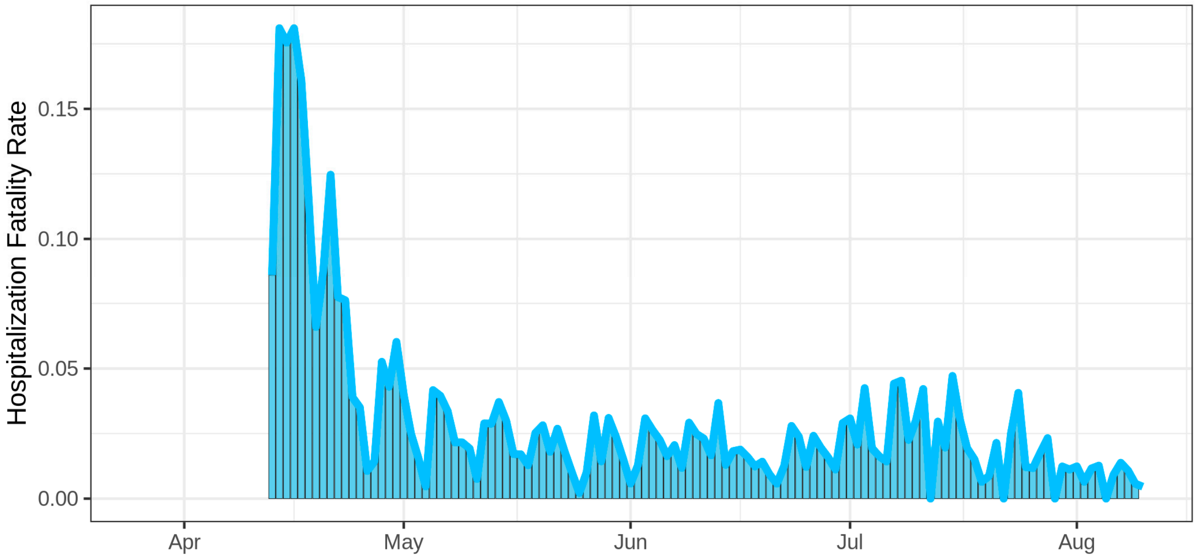

Figure S5. The approximated treatment/hospitalization fatality rate (T/HFR) in New Jersey, adjusted by a delay of ~18 days from hospitalization to death (Table S1). In April, a relatively large portion of deaths were probably taking place in long term care facilities instead of in hospitals.

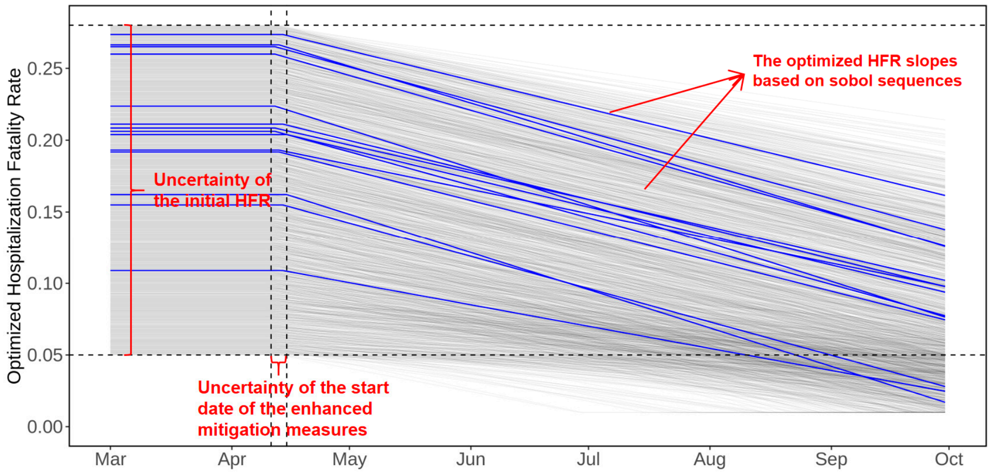

Figure S6. Illustration of the selection process for the estimated treatment/hospitalization fatality rates. The gray lines represent 2,000 T/HFR slopes optimized based on the 2,000 parameter sets (i.e., "low discrepancy" Sobol sequences), the blue lines represent the 14 selected T/HFR slopes optimized based on the 14 "most suitable" sobol sequences.

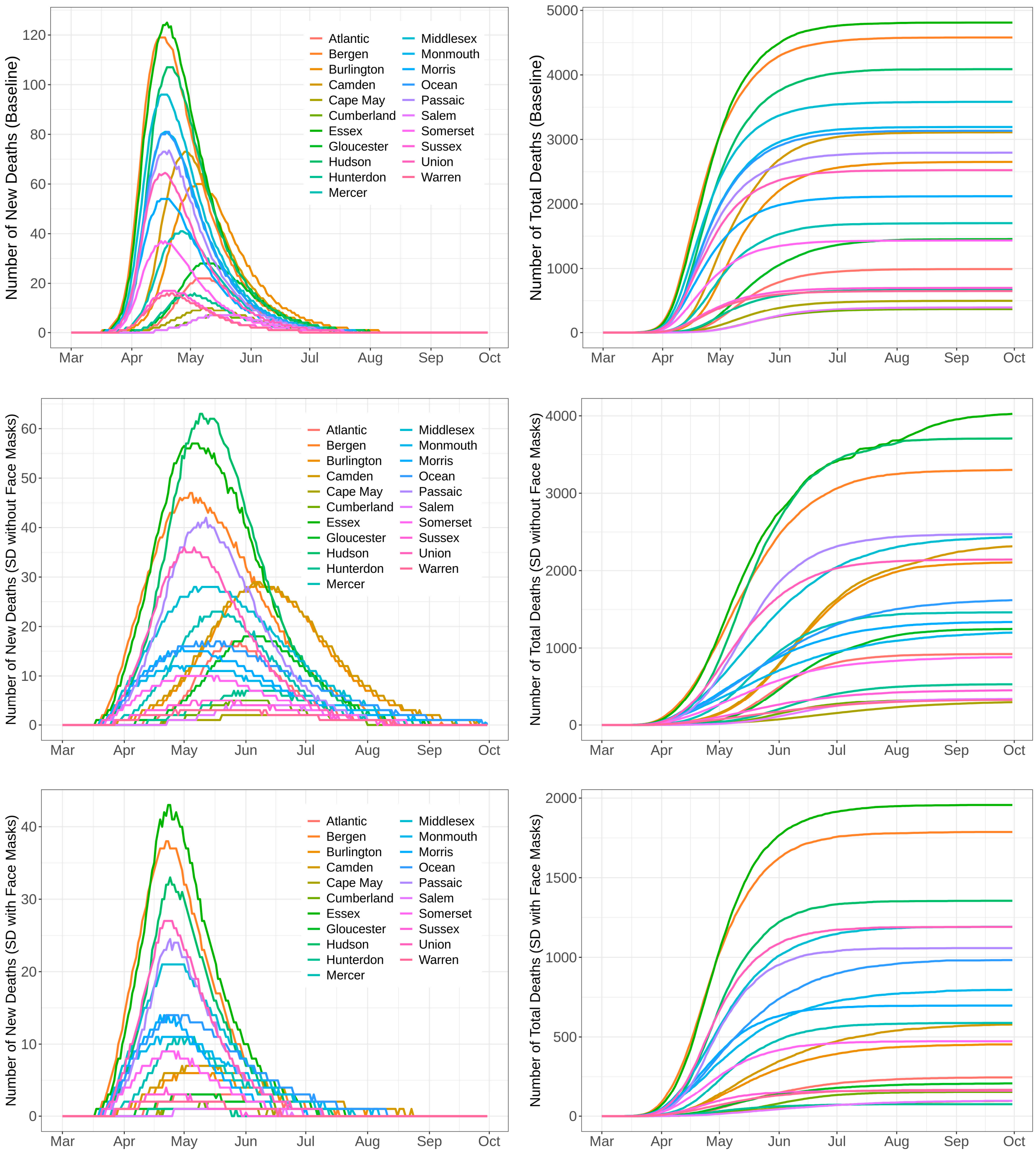

Figure S7. Projected median of new/total deaths from COVID-19 for three exposure scenarios (one factual and two counter-factuals) across the 21 New Jersey counties.

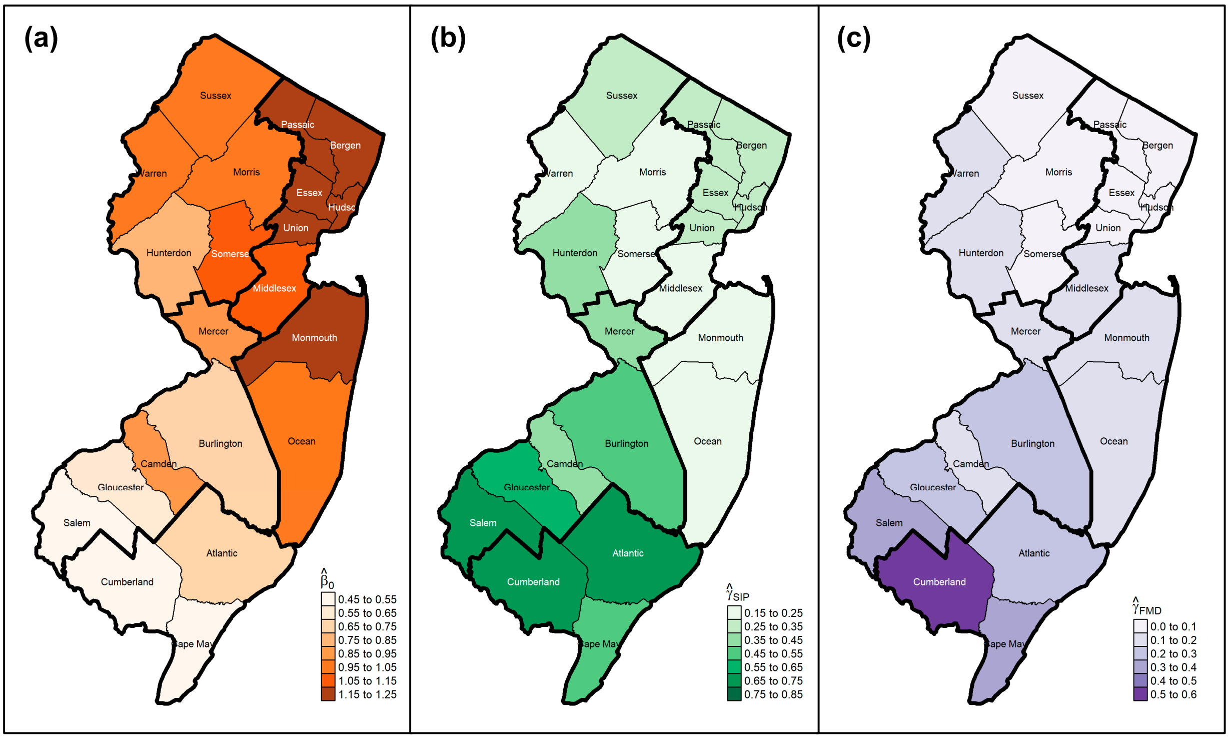

Figure S8. Spatial distribution of the median estimates for the (a) basic transmission parameter β_0, (b) shelter-in-place transmission scaling factor γ_{SIP}, and (c) face masks directive transmission scaling factor γ_{FMD} across the 21 New Jersey counties.

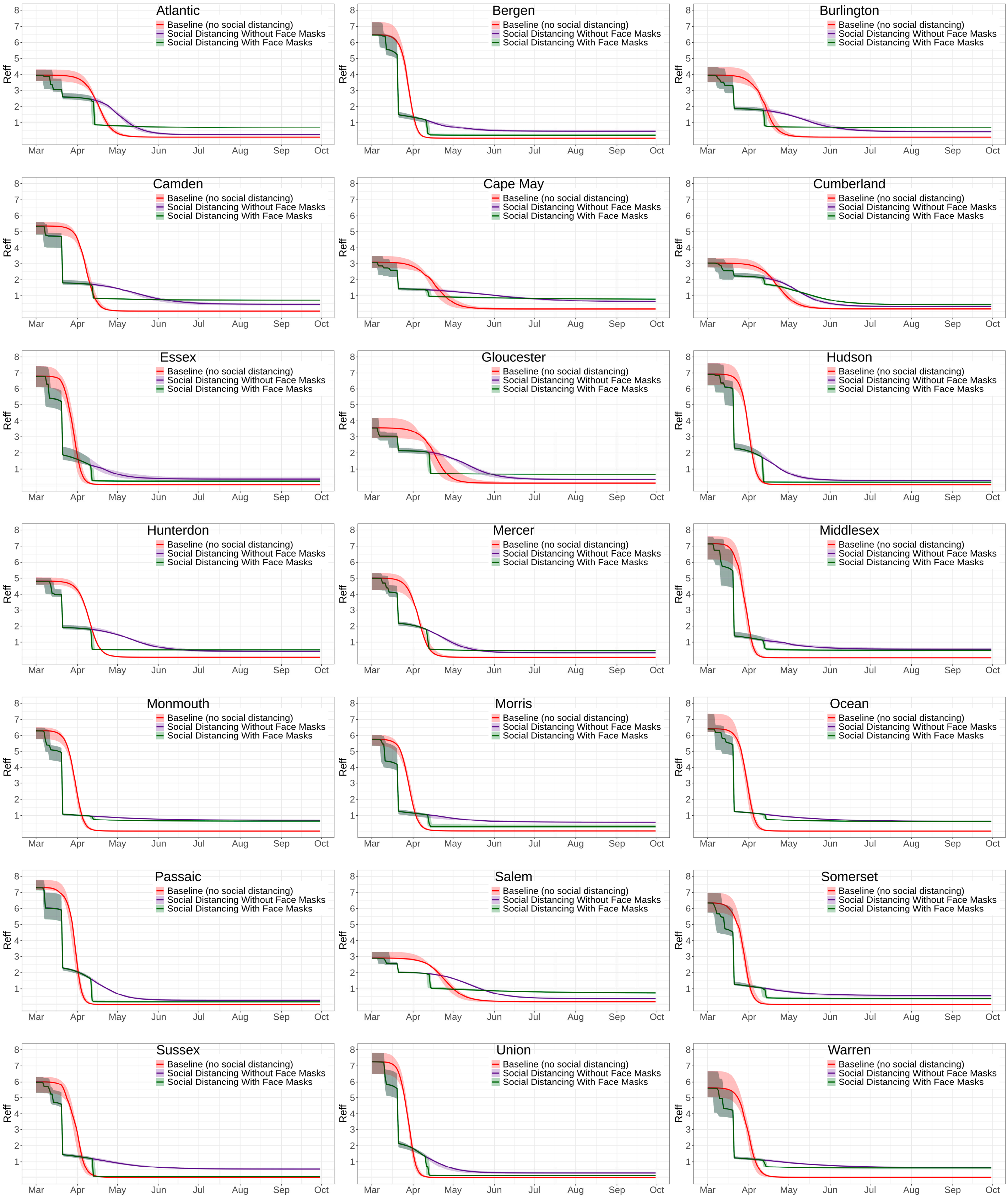

Figure S9. Time-varying effective reproduction number (R_{eff}) for the 21 New Jersey counties in three scenarios.

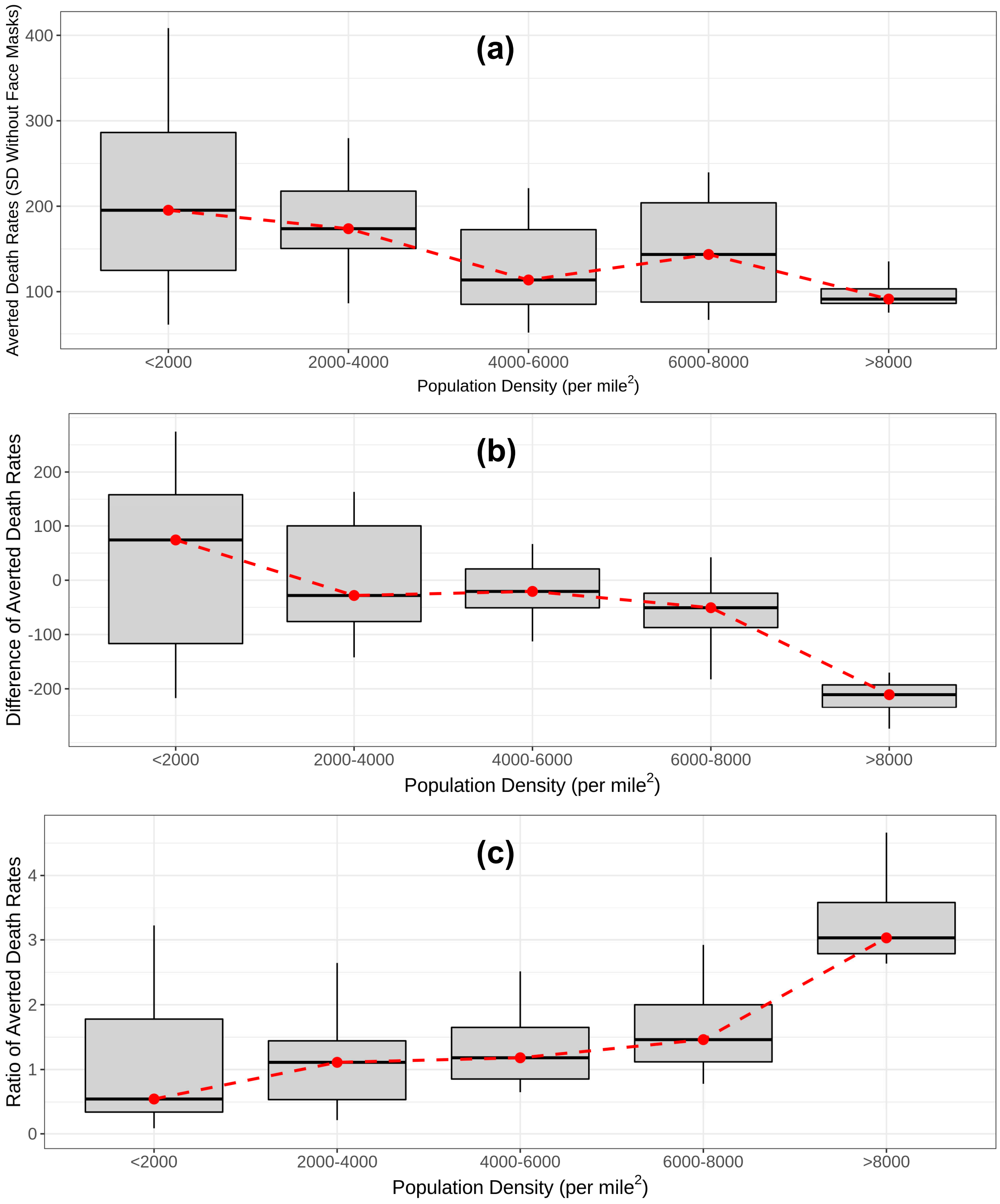

Figure S10. Effectiveness of social distancing and wearing face masks with respect to population density in five categories. The effectiveness of social distancing is quantified via (a) the averted deaths per 100,000 people for scenario 2. The relative effectiveness of wearing face masks is quantified via (b) the difference of the averted deaths per 100,000 people between scenario 3 and scenario 2, and (c) the corresponding ratio metric.

Figure S11. Screenshots of different tabs of the dashboard for the COVID-19 project: https://ccl-eohsi.shinyapps.io/covid19_dashboard/

Figure S11(a). Overview Tab

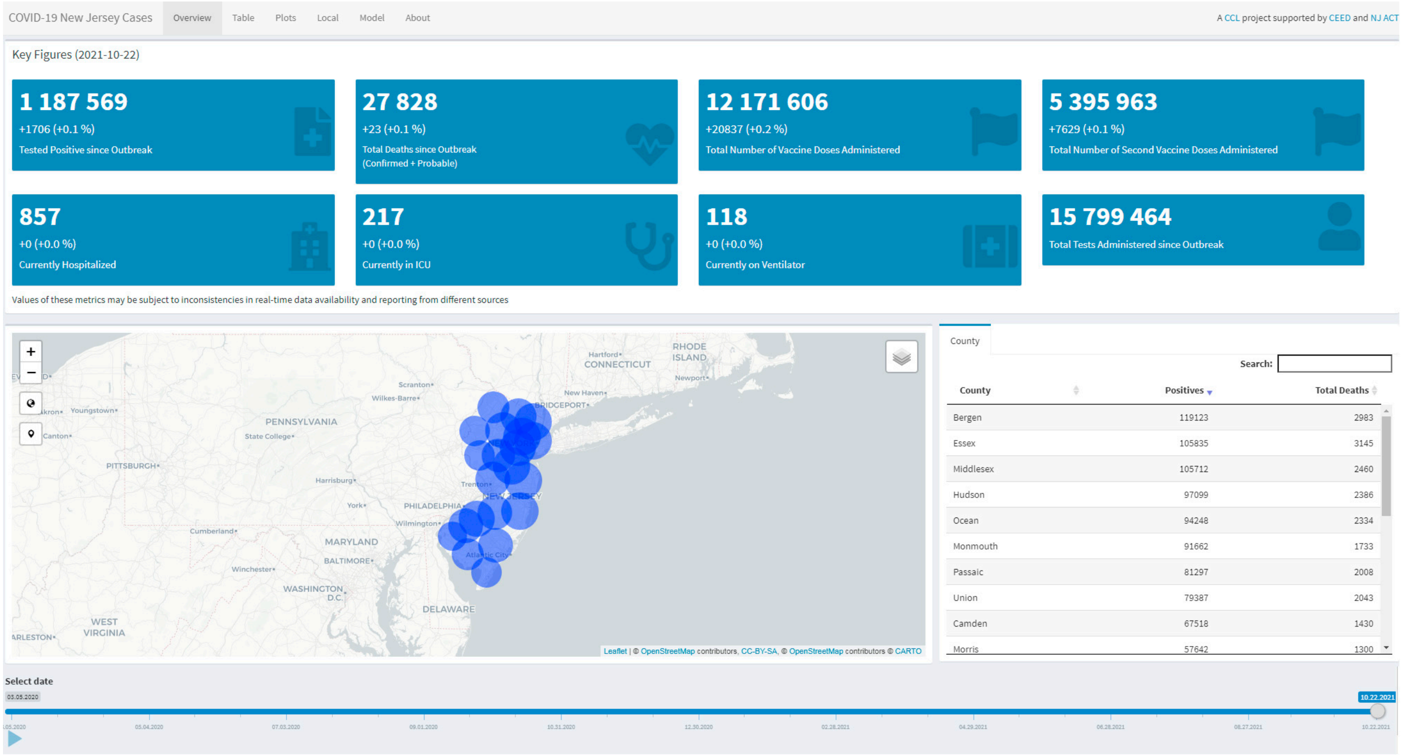

Figure S11(b). Table Tab

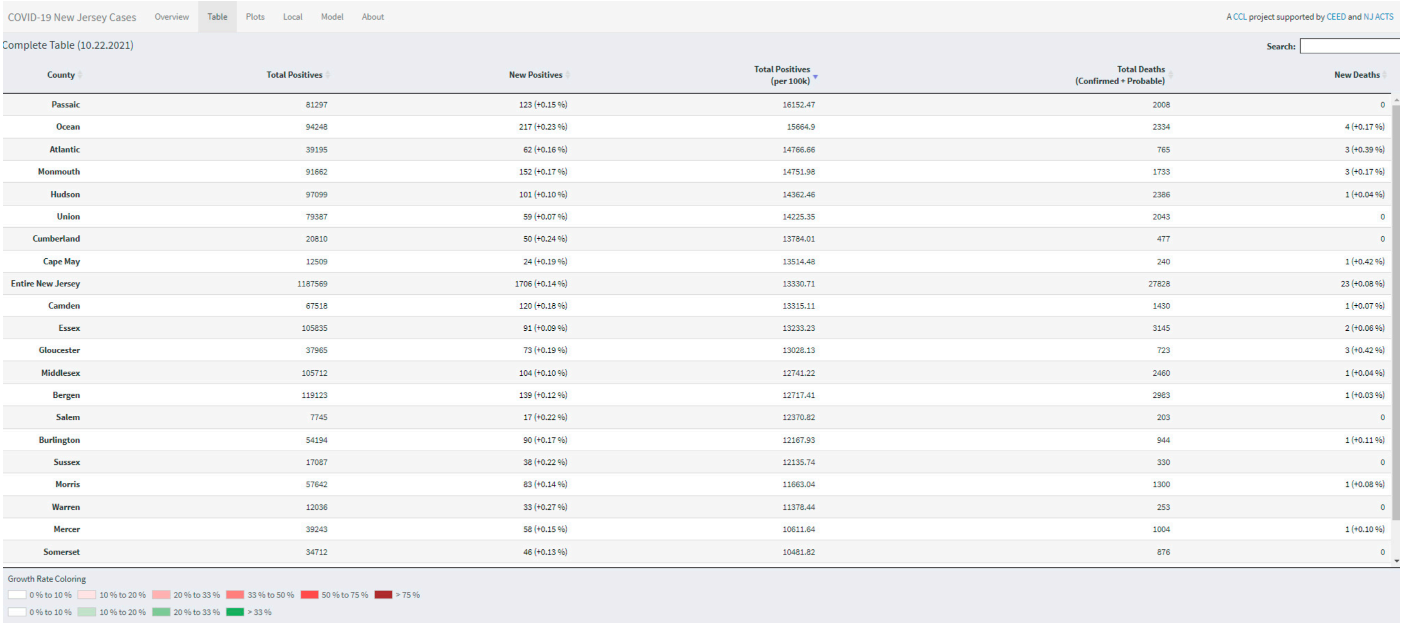

County	Total Positives	New Positives	Total Positives (per 100k)	Total Deaths (Confirmed + Probable)	New Deaths
Passaic	81297	123 (+0.15 %)	16152.47	2008	0
Ocean	94248	217 (+0.23 %)	15664.9	2334	4 (+0.17 %)
Atlantic	39195	62 (+0.16 %)	14766.66	765	3 (+0.39 %)
Monmouth	91662	152 (+0.17 %)	14751.98	1733	3 (+0.17 %)
Hudson	97099	101 (+0.10 %)	14362.46	2386	1 (+0.04 %)
Union	79387	59 (+0.07 %)	14225.35	2043	0
Cumberland	20810	50 (+0.24 %)	13784.01	477	0
Cape May	12509	24 (+0.19 %)	13514.48	240	1 (+0.42 %)
Entire New Jersey	1187569	1706 (+0.14 %)	13330.71	27828	23 (+0.08 %)
Camden	67518	120 (+0.18 %)	13315.11	1430	1 (+0.07 %)
Essex	105835	91 (+0.09 %)	13233.23	3145	2 (+0.06 %)
Gloucester	37965	73 (+0.19 %)	13028.13	723	3 (+0.42 %)
Middlesex	105712	104 (+0.10 %)	12741.22	2460	1 (+0.04 %)
Bergen	119123	139 (+0.12 %)	12717.41	2983	1 (+0.03 %)
Salem	7745	17 (+0.22 %)	12370.82	203	0
Burlington	54194	90 (+0.17 %)	12167.93	944	1 (+0.11 %)
Sussex	17087	38 (+0.22 %)	12135.74	330	0
Morris	57642	83 (+0.14 %)	11663.04	1300	1 (+0.08 %)
Warren	12036	33 (+0.27 %)	11378.44	253	0
Mercer	39243	58 (+0.15 %)	10611.64	1004	1 (+0.10 %)
Somerset	34712	46 (+0.13 %)	10481.82	876	0

Growth Rate Coloring
0 % to 10 % | 10 % to 20 % | 20 % to 33 % | 33 % to 50 % | 50 % to 75 % | > 75 %
0 % to 10 % | 10 % to 20 % | 20 % to 33 % | > 33 %

Figure S11(c). Plots Tab

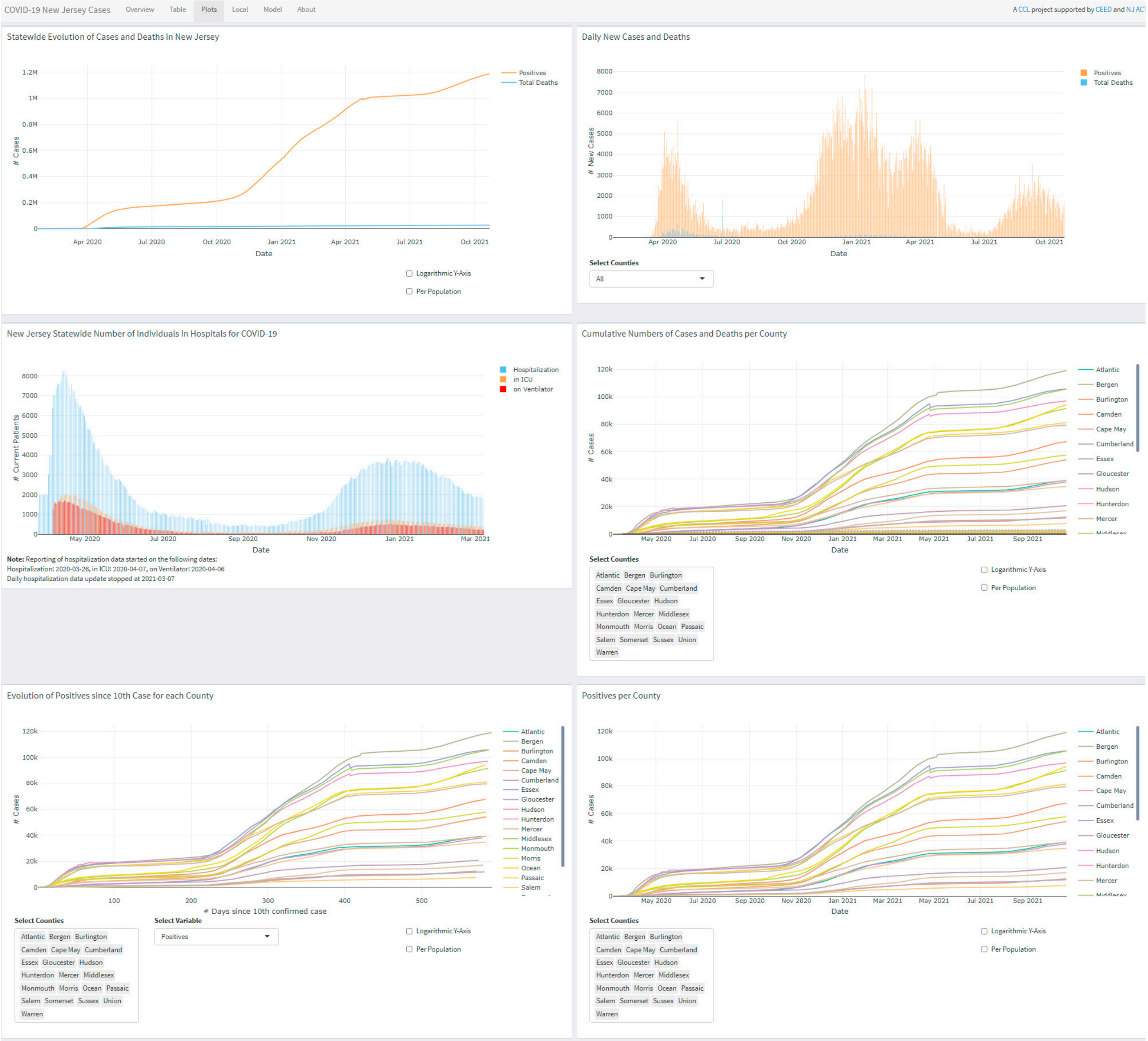

Figure S11(d). Local Tab

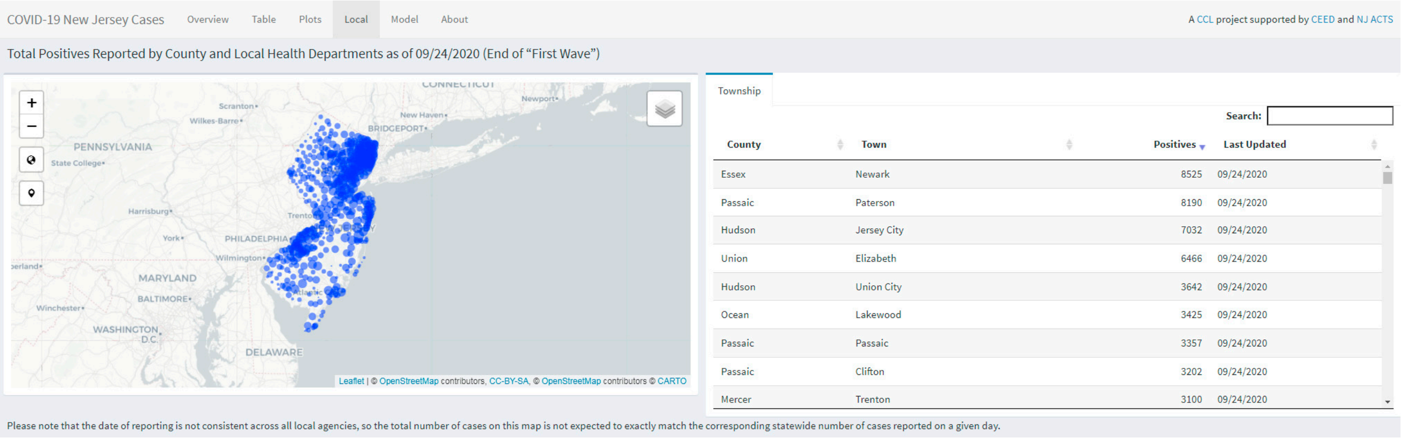

County	Town	Positives	Last Updated
Essex	Newark	8525	09/24/2020
Passaic	Paterson	8190	09/24/2020
Hudson	Jersey City	7032	09/24/2020
Union	Elizabeth	6466	09/24/2020
Hudson	Union City	3642	09/24/2020
Ocean	Lakewood	3425	09/24/2020
Passaic	Passaic	3357	09/24/2020
Passaic	Clifton	3202	09/24/2020
Mercer	Trenton	3100	09/24/2020

Please note that the date of reporting is not consistent across all local agencies, so the total number of cases on this map is not expected to exactly match the corresponding statewide number of cases reported on a given day.

Figure S11(e). Model Tab

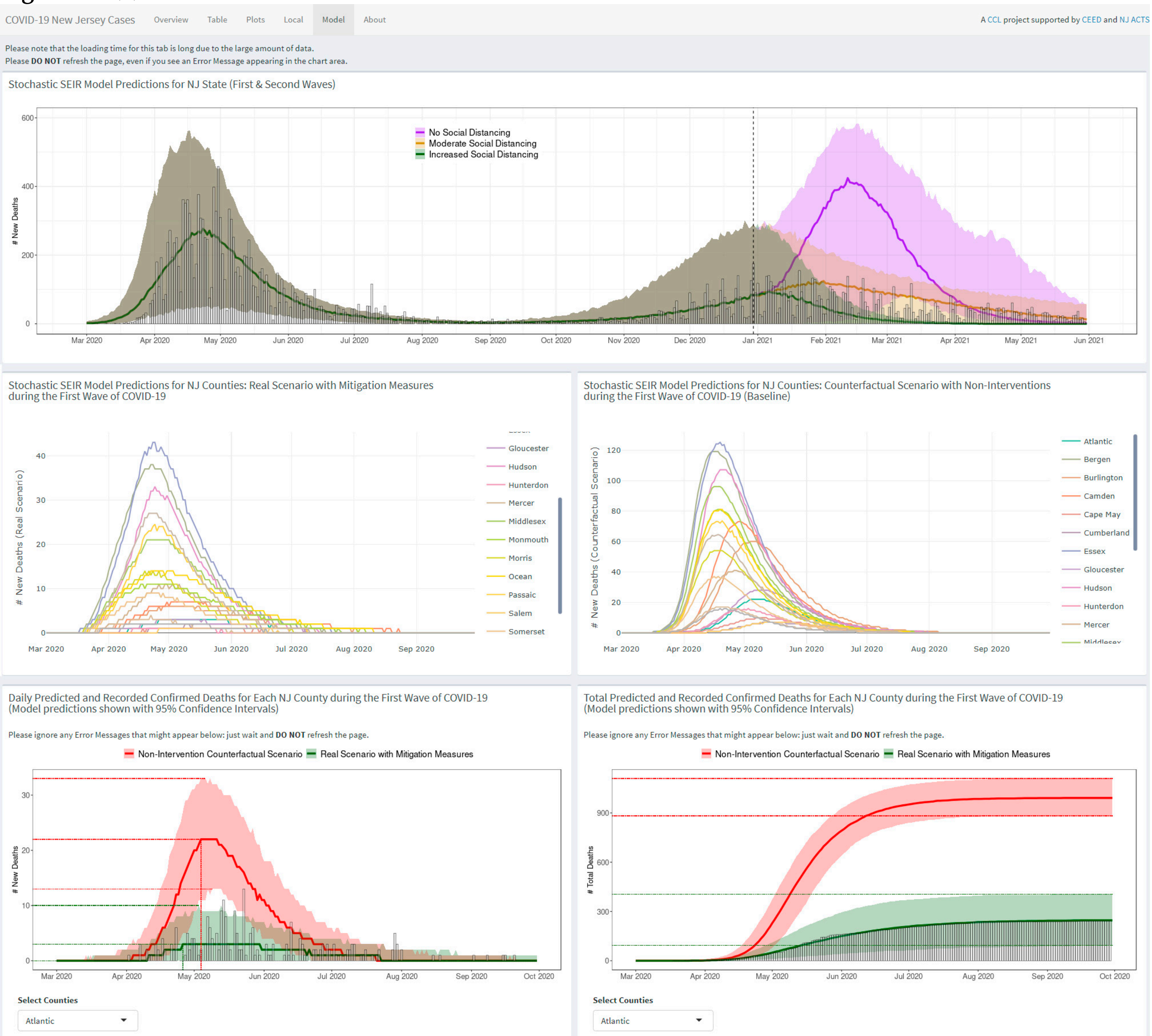

Figure S11(f). About Tab

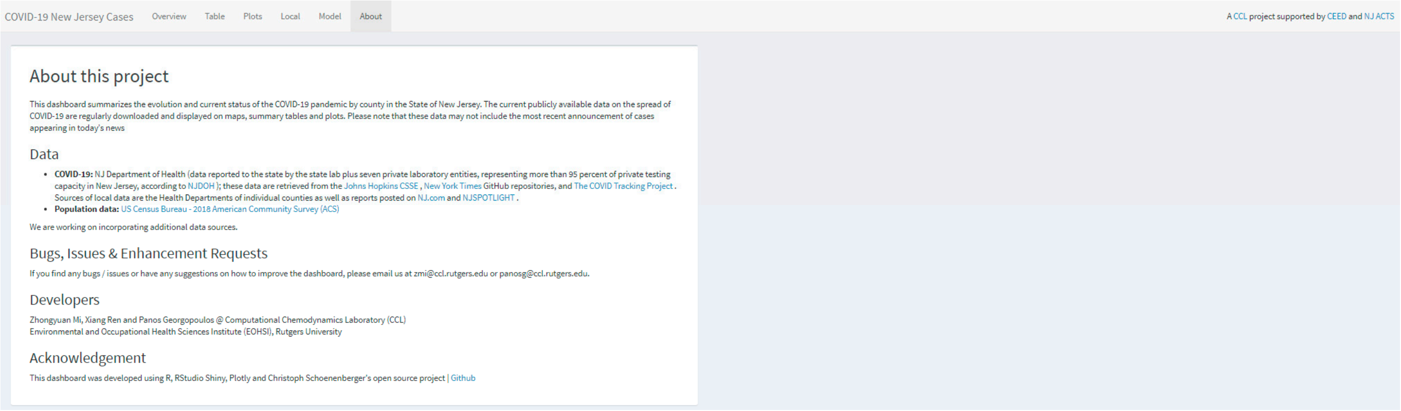

Supplement: Supplementary file 1 [file ijerph-18-11950-s001.zip › ijerph-1430668-supplementary.pdf]
